# Flexible, high mobility short-channel organic thin film transistors and logic circuits based on 4H–21DNTT

**DOI:** 10.1038/s41598-021-91239-7

**Published:** 2021-06-03

**Authors:** Anubha Bilgaiyan, Seung-Il Cho, Miho Abiko, Kaori Watanabe, Makoto Mizukami

**Affiliations:** Innovation Center for Organic Electronics, Yamagata University, 1-808-48, Arcadia, Yonezawa, Yamagata 992-0119 Japan

**Keywords:** Engineering, Materials science, Physics

## Abstract

The low mobility and large contact resistance in organic thin-film transistors (OTFTs) are the two major limiting factors in the development of high-performance organic logic circuits. Here, solution-processed high-performance OTFTs and circuits are reported with a polymeric gate dielectric and 6,6 bis (trans-4-butylcyclohexyl)-dinaphtho[2,1-b:2,1-f]thieno[3,2-b]thiophene (4H–21DNTT) for the organic semiconducting layer. By optimizing and controlling the fabrication conditions, a high saturation mobility of 8.8 cm^2^ V^−1^ s^−1^ was demonstrated as well as large on/off ratios (> 10^6^) for relatively short channel lengths of 15 μm and an average carrier mobility of 10.5 cm^2^ V^−1^ s^−1^ for long channel length OTFTs (> 50 μm). The pseudo-CMOS inverter circuit with a channel length of 15 μm exhibited sharp switching characteristics with a high signal gain of 31.5 at a supply voltage of 20 V. In addition to the inverter circuit, NAND logic circuits were further investigated, which also exhibited remarkable logic characteristics, with a high gain, an operating frequency of 5 kHz, and a short propagation delay of 22.1 μs. The uniform and reproducible performance of 4H–21DNTT OTFTs show potential for large-area, low-cost real-world applications on industry-compatible bottom-contact substrates.

## Introduction

Flexible electronics are emerging as a multi-billion dollar industry with a growing number of applications in various fields of smart electronics such as wearable sensors, implantable biomedical devices, health monitors, and flexible displays^1–6^. Organic thin film transistors (OTFT) are considered one of the most competent candidates for applications in flexible integrated circuits because of their compatibility with low-cost and low-temperature processing techniques, which are crucial for flexible substrates^5–9^. In recent years, research in OTFT technology has led to considerable improvements in OTFT parameters such as charge-carrier mobility (μ), contact resistance (R_C_), and transistor scaling^10–20^. However, to further advance OTFT-based technology for logic circuit applications, enhancement in OTFT parameters and reproducibility and uniformity of device performance is essential in order to demonstrate that sufficient performance advantages can be offered at the device and circuit level compared with TFT counterparts.

Dinaphtho[2,3-b:2,3-f]thieno[3,2-b]thiophene (DNTT) and its derivatives are a promising class of air-stable conjugated semiconductors with large ionization potentials (5.3–5.7 eV) and good mobility. In previous studies, soluble derivatives of DNTT, such as the alkylated DNTT derivative C10-DNTT (2,9-di-decyl-dinaphtho-[2,3-b:20,30;-f]-thieno-[3,2-b]-thiophene), have demonstrated a large crystalline domain using solution-processed techniques such as meniscus guided coating, bar coating, and solution shearing edge casting, leading to OTFT devices with hole mobilities up to 12 cm^2^ V^−1^ s^−1^^13,21–24^. Recently, our group reported a new cyclopentyl-substituted DNTT (5H–21DNTT) on a glass substrate with a hole mobility higher than 15 cm^2^ V^−1^ s^−1^ and a 100% reliability factor^13^. In this work, we first studied the potential of another derivative of DNTT, 6,6 bis (trans-4-butylcyclohexyl)-dinaphtho[2,1-b:2,1-f]thieno[3,2-b]thiophene (4H–21DNTT), which was deposited using a solution-shearing process on both flexible and rigid glass substrates with a polymeric gate dielectric to study the influence of substrates on the OTFT performance. Further, to achieve high-frequency circuit operation, it is necessary to downscale the device dimensions, and contact resistance is an important bottleneck limiting OTFT performance for short-channel devices^10–12^. For large-area and complex circuit designs, inverted (bottom-gate) device architectures and coplanar (bottom-contact) (BG-BC) OTFTs are more favorable because BG-BC design facilitates fabrication and patterning. The contact resistance is typically higher for bottom contact (BC) devices, as gate field-assisted charge injection is weakened^25–27^. We analyzed 4H–21DNTT OTFT performance for different channel lengths, and the contact resistance was estimated using the transmission line method (TLM).

Conventional CMOS logic design requires both n- and p-type devices. For this reason, it is not optimal for OTFT-based circuits because the performance of n-type and p-type OTFTs vary too much in terms of air and bias stability, mobility, and other performance parameters. Therefore, for this study, a zero-VGS load pseudo-CMOS logic design style was used to fabricate pseudo-CMOS inverter and NAND logic circuits^28–30^. To realize practical circuit applications using many OTFT devices, it is essential to achieve both high field-effect mobility and uniform electrical characteristics. Although for solution-processable devices, it is challenging to control parameter variation, in this work we report that optimized solution shearing leads to uniform crystalline films of 4H–21DNTT. This allowed us to achieve an average mobility of 6.4 cm^2^ V^−1^ s^−1^ with a small standard deviation of 1.2 for a 15 μm channel length (L) BG-BC OTFT (Fig. 5e). Here, we further demonstrate the capabilities of our high-mobility 4H–21DNTT OTFTs with low contact resistance to enhance the performance characteristics of logic circuits. The inverter and NAND logic circuits were fabricated on glass and flexible substrates using solution sheared highly uniform and crystalline 4H–21DNTT films. Furthermore, this combination of high-performance short-channel devices with small gate-to-contact overlap (L_OV_) and optimized fabrication conditions was instrumental in achieving improved organic logic circuit performance. The inverter logic circuit reached gain and propagation delay of 31.5 μs and 6.9 μs respectively. The NAND logic circuit demonstrated sharp input–output characteristics and stable operation at a frequency of 5 kHz and a V_DD_ of 20 V.

## Results

### OTFT devices with solution processed 4H–21DNTT films

**Figure 1:**
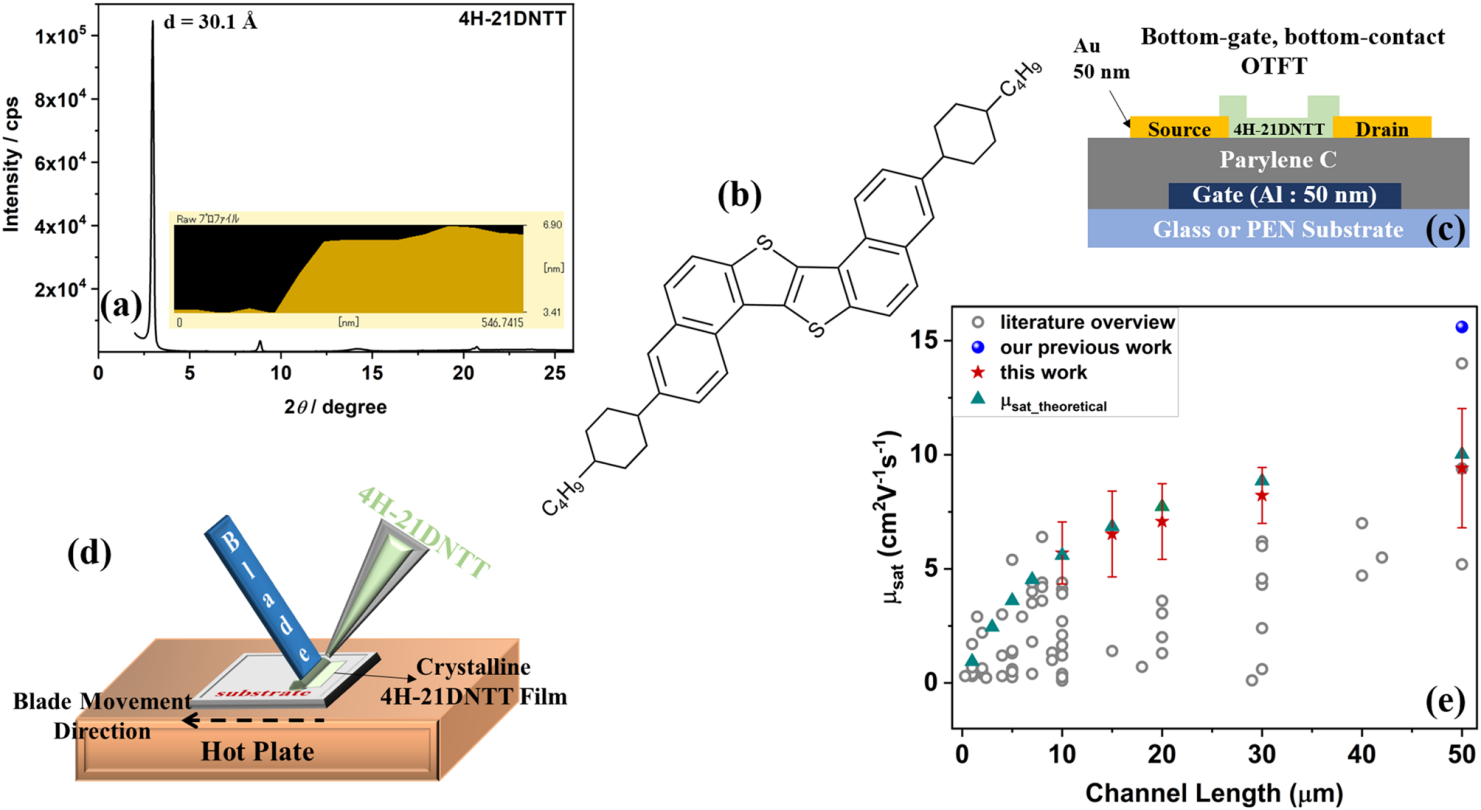
(**a**) Out of plane XRD profile of 4H–21DNTT films on Parylene C/glass substrate fabricated by solution shearing and cross-sectional AFM profile of one monolayer of 4H–21DNTT film (inset). (**b**) Molecular structure of 4H–21DNTT. (**c**) Device Schematic of 4H–21DNTT OTFT with bottom-gate, bottom-contact architecture. (**d**) Schematic illustration of solution shearing set-up. (**e**) Literature overview of OTFT effective mobility for short channel length devices for channel length in range of 300 nm–50 μm^12–20,41–43,45–65^ and for 4H–21DNTT OTFT theoretical effective mobility (μ_sat_theoretical_) calculation; μ_int_avg_ = 12.5 cm^2^ V^−1^ s^−1^ and R_c_W = 1 kΩcm.

**Figure 2:**
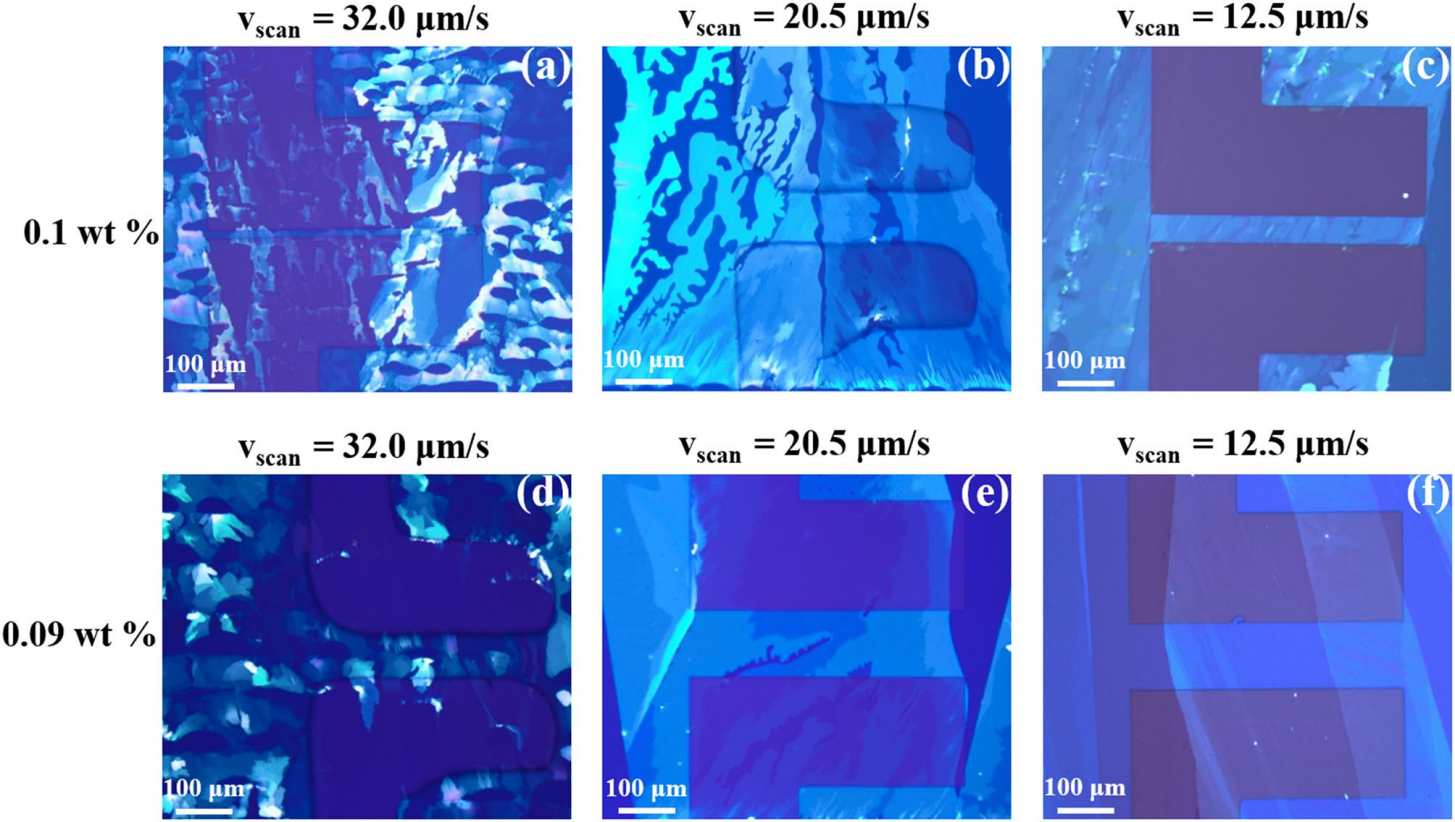
Characterization of 4H–21DNTT films deposited from solution shearing at different blade scanning speed (**a**–**c**) Polarized microscopy images of 4H–21DNTT film at 0.1 wt% concentration in o-DCB solution (**d**–**f**) Polarized microscopy images of 4H–21DNTT film at 0.09 wt% concentration in o-DCB solution.

For this work, as illustrated in Fig. 1c, bottom-gate bottom-contact (BG-BC) OTFTs were fabricated on both rigid glass and 125-μm thick flexible polyethylene naphthalate (PEN) substrates. Patterned aluminum (50 nm) gate electrodes were deposited on cleaned substrates through a shadow mask. Parylene C was selected as the organic gate dielectric layer because it is well known for its environmental stability, low dielectric constant, low defect density, and optimal surface energy for solution shearing with many solvents, and hence is an appropriate selection for bottom gate OTFT configuration^13,31,32^. Parylene C was deposited using chemical vapor deposition (CVD) on substrates with patterned gate electrodes. Independently, the relative dielectric constant for Parylene C was estimated to be *ε*_r_ = 3.1 from capacitance measurements on a metal insulator metal (MIM) device structure. Au electrodes were deposited through a shadow mask with a channel width (W) of 380–400 μm and various channel lengths (L) ranging from 10 to 150 μm. In order to facilitate charge injection by modification of the contact work function the gold electrodes were treated with 2,3,4,5,6-pentafluorothiophenol (PFBT) thiol-SAM prior to semiconductor deposition. A thin film of 4H–21DNTT semiconductor (Fig. 1b) was deposited using a solution shearing process with a thin glass blade (the schematic of the OSC deposition process is shown in Fig. 1d). The glass blade was coated with a thin Teflon layer to make the surface hydrophobic; this prevents OSC from adhering to the blade during solution shearing. In solution shearing technique the deposition parameters such as substrate temperature, concentration of organic semiconductor (OSC) and blade speed have a major influence on the crystalline film formation. Previous reports on optimization of process parameters for meniscus-guided coating techniques have shown that to achieve highly crystalline film the optimal coating speed depends on the substrate temperature and the choice of OSC/solvent system^33–38^. In this work, for 4H–21DNTT film optimization we used two concentrations of 4H–21DNTT (0.1 wt% and 0.09 wt%) in o-dichlorobenzene (o-DCB) solvent. The small amount of 4H–21DNTT solution (6 μL) was inserted in the small gap (∼ 100–200 μm) between blade and Parylene C coated substrates (shearing setup in Fig. 1d). 4H–21DNTT film was deposited at 90 ℃ substrate temperature for different blade scanning speeds. Figure 2 shows the polarized microscope images of 4H–21DNTT film under different processing conditions. All images were taken under same light intensity therefore the film brightness suggests the thickness of the film. At higher coating speeds voids are observed in the film due to faster scanning speed than the rate of solvent evaporation^37^. The voids size is reduced as the coating speed decreases and matches closely with solvent evaporation rate, at a blade speed of 12.5 μm/s highly ordered 4H–21DNTT film was obtained. For higher solute concentration the 4H–21DNTT film thickness was increased and thick aggregates were formed in the film (Fig. S3). The thick aggregates in the semiconductor film are undesirable as it can lower the performance of OTFT device^34^. The OTFT characteristics for various OSC deposition condition (Fig. S2) reflects the OSC morphology dependence. The OTFT devices for films with thick aggregates and voids showed poor performance and lower mobility. The solution shearing speed was found to be very critical in achieving the good OTFT performance and void free crystalline 4H–21DNTT film. The best optimized condition (0.09 wt% concentration and 12.5 μm/s blade speed) was selected to achieve a uniform single-crystal 4H–21DNTT thin film with a thickness of a few molecular layers to further investigate the OTFT performance from here on.

The solution-sheared 4H–21DNTT thin film on Parylene C-coated glass substrate was investigated by X-ray diffraction (XRD) to better understand its structure and crystallinity (Fig. 1a). The intense and sharp peaks in out-of-plane measurements show the high crystallinity of 4H–21DNTT thin films on the glass/Parylene C substrate. The XRD peaks for 4H–21DNTT can be assigned to (00*l*) reflections. The crystal growth direction corresponds to the crystallographic c-axis of 4H–21DNTT molecules, i.e., nearly perpendicular to the substrate. The interlayer distance (d-spacing) estimated from the XRD data was 30.1 Å for 4H–21DNTT. The thickness of the mono-layer of 4H–21DNTT estimated from the cross-sectional AFM profile (Fig. 1a, inset) was approximately 2.9 nm, which is consistent with the single molecular length estimated from XRD. The 4H–21DNTT exhibited similar properties, such as the XRD profile and absorption spectra (Fig. S1), to those of the previously reported 5H-21DNTT organic semiconductor.

### OTFT device characterization

**Figure 3:**
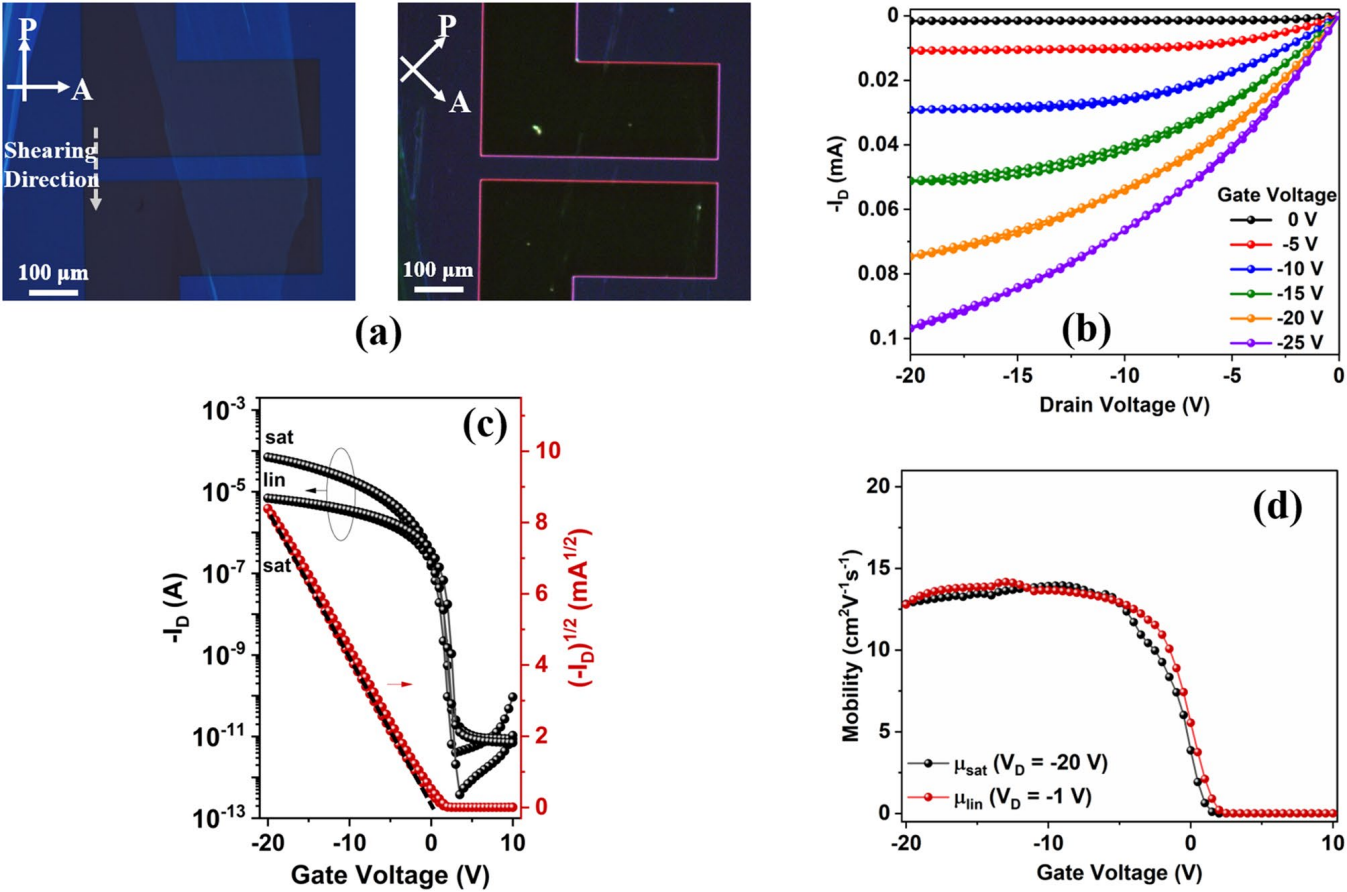
(**a**) Polarized optical microscopy (POM) images of the 4H–21DNTT crystalline film deposited on channel region defined by Au bottom contacts on Parylene C/glass substrate. (**b**) Output characteristics 4H–21DNTT OTFT at different gate bias voltage. (**c**) Transfer characteristics of the 4H–21DNTT OTFT devices (W/L = 400 μm/80 μm) on Parylene C/glass substrate in the saturation region (V_D_ =  − 20 V) *μ*_sat_ = 13.0 cm^2^ V^−1^ s^−1^ and linear region (V_D_ =  − 1 V) *μ*_lin_ = 13.6 cm^2^ V^−1^ s^−1^. (**d**) Saturation and linear mobility vs gate voltage plot for 4H–21DNTT OTFT prepared by shearing on Parylene C/glass substrate.

**Figure 4:**
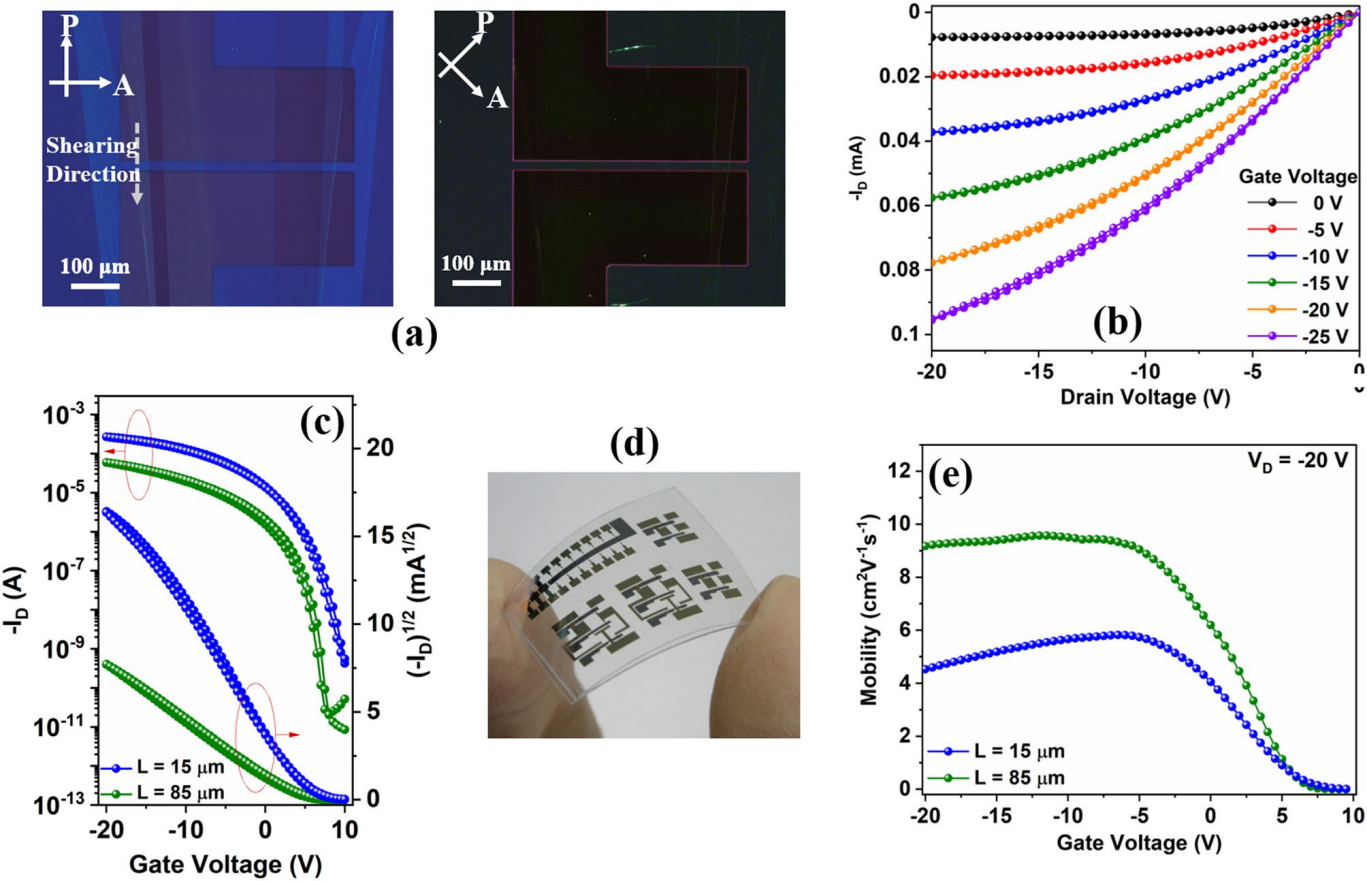
(**a**) Polarized optical microscopy (POM) images of the 4H–21DNTT crystalline film deposited on channel region defined by Au bottom contacts on Parylene C/flexible (PEN) substrate. (**b**) Output characteristics 4H–21DNTT OTFT at different gate bias voltage. (**c**) Transfer characteristics of the 4H–21DNTT OTFT devices (W/L = 450 μm/85 μm and 15 μm) on Parylene C/ flexible (PEN) substrate in the saturation region (V_D_ =  − 20 V) *μ*_sat_ (L = 85 μm) = 9.40 cm^2^ V^−1^ s^−1^ and *μ*_sat_ (L = 15 μm) = 6.33 cm^2^ V^−1^ s^−1^. (**d**) Photograph of OTFT fabricated on flexible PEN substrate. (**e**) Saturation mobility (L = 85 μm and 15 μm) vs gate voltage plot for 4H–21DNTT OTFT prepared by shearing on Parylene C/flexible (PEN) substrate.

Figure 3a shows a cross-polarized optical microscope image of 4H–21DNTT on BG-BC OTFT on glass substrate, showing that the large single-crystal morphology of 4H–21DNTT. On rotating the polarizer and analyzer angle by 45°, completely black images were obtained, indicating that the 4H–21DNTT films had good crystallinity and were uniformly oriented. The electrical performance characteristics of 4H–21DNTT BG-BC OTFT fabricated on a glass substrate are summarized in Fig. 3. The surface around the channel region was scratched mechanically to reduce the possibility of fringe currents^39^, and all the measurements were performed at room temperature (25 ℃ to 27 ℃), in air, and under dark conditions. Figure 3b shows the output characteristics (I_D_ vs. V_D_) for different gate voltages of the 4H–21DNTT OTFT with channel length (L) 80 μm and width (W) 400 μm. Figure 3c shows the transfer characteristics of the 4H–21DNTT OTFT (W/L = 400/80) in the saturation regime (V_D_ =  − 20 V) and linear regime (V_D_ =  − 1 V); negligible hysteresis was observed in both the output and transfer curves. The saturation (μ_sat_) and linear (μ_lin_) mobilities were calculated from the slopes of the fitted lines for the plots of (I_D_)^1/2^ vs. V_G_ and I_D_ vs. V_G_, respectively. For the 4H–21DNTT OTFT device (Fig. 3c), the on/off ratio was greater than 10^7^ and in the saturation region (V_DS_ =  − 20 V) the mobility (μ_sat_) is estimated to be 13.0 cm^2^ V^−1^ s^–1^ with 99.6% reliability factor (r)^40^ and a near zero threshold voltage (V_TH_) of 0.4 V. In the linear region (V_DS_ =  − 1 V) mobility (μ_lin_) was estimated to be 13.6 cm^2^ V^−1^ s^–1^ with 98.2% reliability factor (r) and a threshold voltage (V_TH_) of 0.2 V. A plot of mobility (in the saturation and linear regions) vs. gate voltage (Fig. 3d) shows a broad flat region over a large gate voltage range, which indicates hardly any nonlinear contact issues. Furthermore, the gate-to-source leakage current (I_GS_) was less than 10^−10^ A. The sub-threshold swing (SS) was determined to be 158.5 ± 3 mV/decade by fitting the exponential region of the drain current in the sub-threshold regime (Fig. 3b). Figure S8 shows the electrical characteristics of the 4H–21DNTT OTFT transistor after the substrate had been exposed to ambient air and room temperature for 30 days. The aged device has shown only 14.1% decrease in mobility and a threshold voltage shift of 0.08 V after 30 days of continuous exposure to air and light. Figure 4a shows a cross-polarized optical microscope image of 4H–21DNTT on BG-BC OTFT on flexible (PEN) substrate, showing that the large single-crystal morphology of 4H–21DNTT is very similar regardless of the type of substrate (glass or PEN). Figure 4b,c show the output and transfer curves of 4H–21DNTT OTFT fabricated on the flexible PEN substrate. The flexible OTFT devices (photograph in Fig. 4d) demonstrate negligible hysteresis, good switching behavior with high on–off ratios (> 10^7^), onset voltages close to 0 V and nearly gate-independent saturation mobility of 9.4 cm^2^ V^−1^ s^–1^ for long channel (85 μm) and 6.33 cm^2^ V^−1^ s^–1^ for short channel (15 μm) devices as shown in Fig. 4e.

### High mobility short channel OTFT and contact resistance analysis

The output characteristics do not show any apparent nonlinearity at small V_DS_ voltages, which suggests that the contact resistance of the 4H–21DNTT OTFT device type is low. However, the contact effects become more pronounced and critical for reducing the channel length. The contact resistance (R_C_) of our bottom-contact OTFTs was evaluated in the linear regime using the transmission line method (TLM). For this purpose, we fabricated 4H–21DNTT OTFT with varying channel lengths ranging from 10 to 150 μm. The total resistance (R_Total_) between the source and drain electrodes in the TLM is defined as the sum of the channel resistance (R_ch_) and contact resistance (R_C_) at the source and drain electrodes^15,16^.

1 $$R_{Total} = R_{ch} + R_{C} = \frac{{\partial V_{D\_lin} }}{{\partial I_{D\_lin} }}.$$

**Figure 5:**
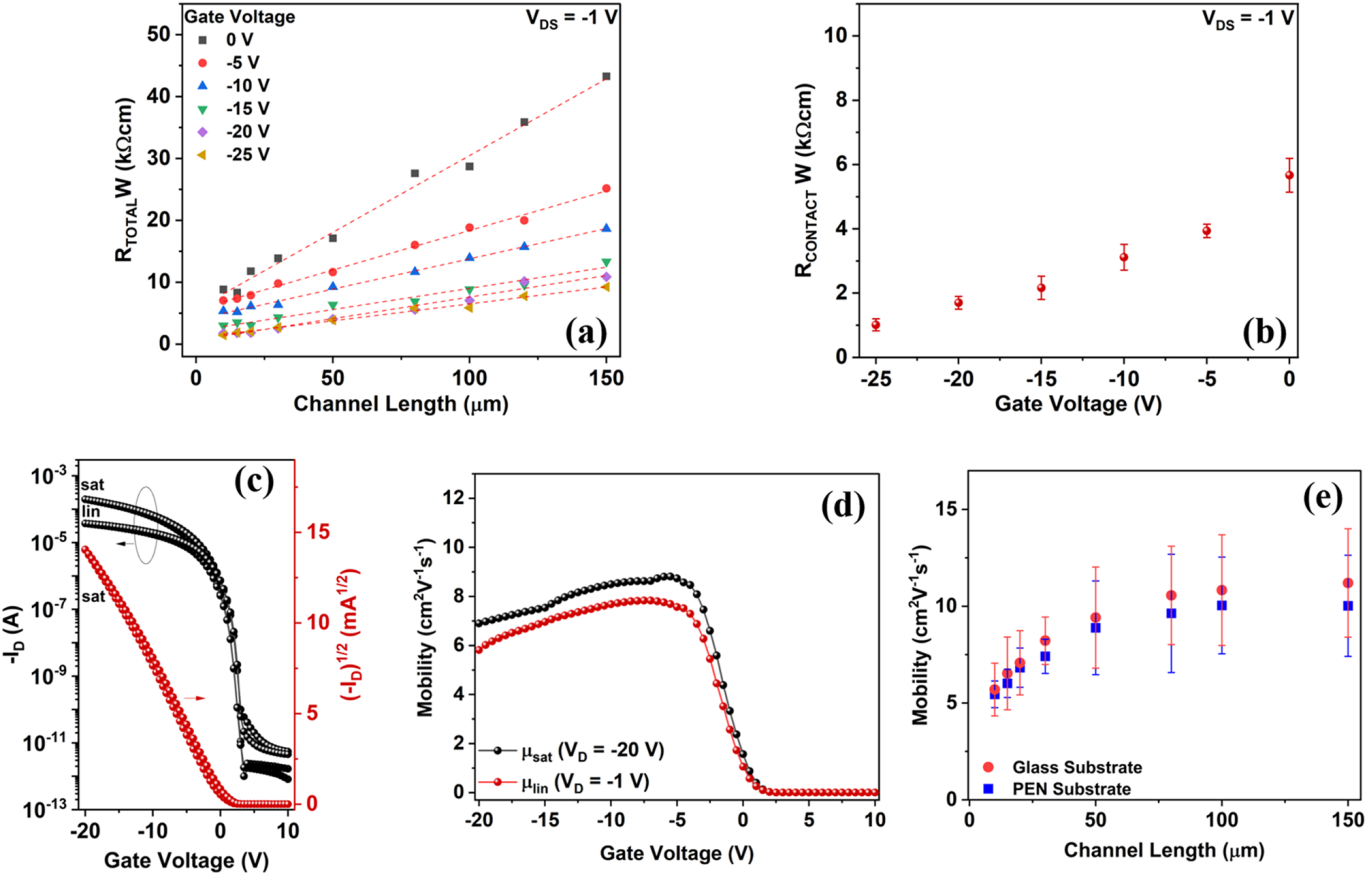
(**a**) Total device resistance (R_TOTAL_W) plotted as a function of the channel length for 4H–21DNTT OTFT for various gate overdrive voltages (*V*_G_ − *V*_TH_). (**b**) Contact resistance calculated using the transmission line method (TLM) plotted as a function of gate overdrive voltages (*V*_G_ − *V*_TH_). (**c**) Transfer characteristics of the 4H–21DNTT OTFT devices (W/L = 450 μm/15 μm) on Parylene C/glass substrate in the saturation region (V_D_ =  − 20 V) *μ*_sat_ = 8.8 cm^2^ V^−1^ s^−1^ and linear region (V_D_ =  − 1 V) *μ*_lin_ = 6.8 cm^2^ V^−1^ s^−1^. (**d**) Saturation and linear mobility vs gate voltage plot for 4H–21DNTT OTFT prepared by shearing on Parylene C/glass substrate. (**e**) Average mobility with standard error vs channel length for 4H–21DNTT OTFT on both glass and flexible (PEN) substrate (For each channel length over 10 devices were used to calculate average mobility).

The contact resistance was estimated from the intercept of the linear fit of the channel width normalized total resistance (R_Total_W) vs. channel length (Fig. 5a) and calculated using the following equation^15,16^:

2 $$R_{Total} \cdot W = R_{C} \cdot W + \frac{L}{{\mu_{o} C_{i} \left( {V_{GS} - V_{th} } \right)}},$$

where μ_o_ is the intrinsic mobility and C_i_ is the insulator capacitance. From the slope of the above equation, the intrinsic mobility (i.e., without the influence of contact resistance) of 4H–21DNTT was estimated. The evolution of the channel width normalized contact resistance (R_C_W) as a function of gate overdrive voltage is plotted in Fig. 5b. The channel width normalized contact resistance (R_C_W) for the 4H–21DNTT device is a minimum of ∼1.01 kΩcm and 2.3 kΩcm at V_G_ of − 20 V for glass and flexible substrates, respectively, and then typically increases with smaller |V_G_|. Despite R_C_*W* values below 100 Ωcm have been demonstrated for BG-BC OTFT with ultra-thin gate dielectric layer^41^, our R_C_*W* values are quite low compared with previously reported for a solution processed BG-BC OTFT with a thick gate dielectric layer^42^. The literature of R_C_W values for coplanar OTFT is shown in Figure S6a. In OTFTs, the energy level misalignment between contacts and OSC is major contributor for higher contact resistance^13,15,43^. The work function of untreated Au contacts was measured to be 4.8 eV which created a hole injection barrier with the HOMO level of 4H–21DNTT (5.31 eV) and resulted in larger contact resistance of 9.4 kΩcm at − 20 V (Fig. S7). For PFBT treated Au contacts the work function was measured to be 5.58 eV, which suggested an efficient charge injection and nearly one order lowering of contact resistance. We concluded that the larger contact resistance with untreated Au contacts is owing to injection barrier due to energetic mismatch, since no major differences were observed in the morphology of the film as a function of PFBT treatment. Another important factor influencing the contact resistance is the morphology of OSC near the contacts as charge injection occurs at the edge of the channel. Several studies have demonstrated that when OSC have non ideal morphology such as large number of small grains and grain boundaries the trap density is significantly increases leading to a higher contact resistance^44^. At the S/D and OSC interface a smooth crystalline morphology of 4H–21DNTT was observed from polarized microscope images (Fig. 3a) and AFM topography images (Fig. S2a); therefore, we speculate that the contact resistance values were not influenced by the traps originating from OSC microstructure. This indicates that the cumulative effect of an improved charge injection due to PFBT-treated S/D electrodes and fewer charge trapping sites at the semiconductor and gate dielectric interface due to highly crystalline and smooth 4H–21DNTT films, contribute to a reduction in the *R*_C_ value and nearly ideal OTFT characteristics. Further reduction of the contact resistance for bottom-contact devices might be possible with very thin gate dielectric layers^12,41,45^, however, discussion on gate dielectric thickness is beyond the scope of this report. The maximum and average intrinsic mobility (μ_o_) for 4H–21DNTT BG-BC OTFT was 15.1 cm^2^ V^−1^ s^−1^ and 12.5 cm^2^ V^−1^ s^−1^ respectively. The μ_o_ for 4H–21DNTT is close to the saturation and linear mobility extracted from the slope of the transfer characteristics because of a relatively low contact resistance in our devices.

In order to address the reproducibility and uniformity of the solution-processed 4H–21DNTT OTFTs, the statistical distribution (standard error) of the extracted mobilities on glass and flexible PEN substrates for each channel length (10–150 μm) are compared in Fig. 5e. For both flexible and glass substrates, the statistics were calculated from more than 10 devices at each channel length. For all channel lengths, the average mobility of 4H–21DNTT devices was greater than 5 cm^2^ V^−1^ s^−1^. The statistical variation of 4H–21DNTT OTFTs with L ranging from 50 to 150 μm for over 50 transistors is shown in Fig. S5. The mean mobility achieved for long channel length devices (L ≥ 50 μm) was 11.1 cm^2^ V^−1^ s^−1^ and a standard deviation of 1.7. The standard deviation in OTFT performance can be further reduced by using fully automated shearing system with precise control over deposition parameters such as flow rate, shape of the meniscus for each device which is not possible with our current available facilities. The drop in mobility with decreasing channel length indicates that the contact resistance effect becomes more pronounced for short channel length devices. The transfer characteristics of a short channel OTFT with a 15 μm channel length is shown in Fig. 5c (transfer characteristics for each channel length are shown in Fig. S4). The mobility (in the saturation and linear regions) vs. gate voltage plot (Fig. 5d) shows a broad flat region with slight gate voltage dependence, which indicates hardly any nonlinear contact issues even for 10 μm channel length. The highest extracted field-effect mobilities for 15 μm and 10 μm channel lengths (L) were 8.8 and 6.9 cm^2^ V^−1^ s^−1^ respectively, the comparison of the 4H–21DNTT field effect mobility for short channel OTFTs (L ≤ 50 μm) with the previously reported results is shown in Fig. 1e. Even with the contact resistance of 1.01 kΩ the mobility value achieved for 4H–21DNTT is comparable to that of the top-performing p-type OTFTs reported in the previous literature^12–20,41–43,45–65^ (as shown in the graph in Fig. 1e). Furthermore, as the transition or cut-off frequency (f_T_) which determines the highest frequency that can be amplified by a single OTFT scales approximately with μ_o_/L^2^^12^, Fig. S6b shows the literature over view of μ_o_/L^2^ values. We believe that the operational frequency performance of 4H–21DNTT devices can be further increased by lowering the R_C_W value, operational voltage and scale the device dimensions which is a challenge for coplanar solution processed OTFTs with organic gate dielectric. However, despite high contact resistance the 4H–21DNTT demonstrated competent short channel OTFT performance.

### Inverter logic circuit performance based on 4H–21DNTT OTFT

**Figure 6:**
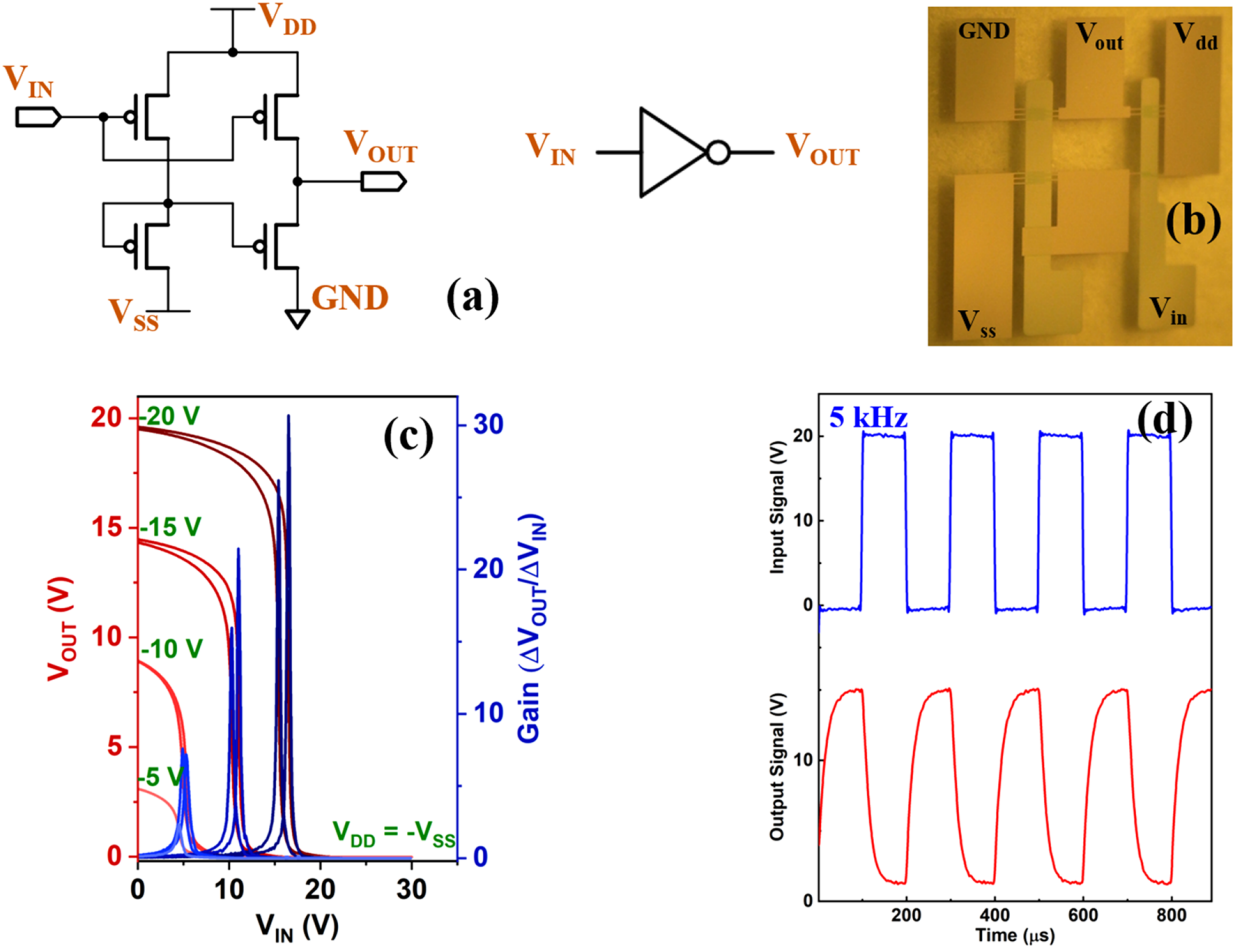
Inverter circuit design and characteristics (**a**) Circuit schematic of zero-V_GS_-load p-type pseudo-CMOS inverter. (**b**) Photograph of fabricated inverter circuit. (**c**) Measured output voltage (V_OUT_) and small-signal gain as a function of input voltage (V_IN_) for supply voltages (V_DD_) between − 5 and − 20 V. (**d**) Dynamic response of zero-V_GS_-load p-type pseudo-CMOS inverter with input signal frequency of 5 kHz.

To further assess the robustness and applicability of our 4H–21DNTT OTFTs in complex integrated circuits, we first evaluated the inverter logic gate, which is the basic component of digital circuits. The modern CMOS design style is unsuitable for OTFT-based circuits because of the large difference in the performance and stability of p-type versus n-type OTFTs. Therefore, for this work, we employed a pseudo-CMOS configuration as it uses unipolar TFTs^28–30,66^. A schematic of the pseudo-CMOS inverter with zero-VGS load logic (pseudo-D) is shown in Fig. 6a. The zero-VGS load logic requires depletion mode TFTs (i.e., normally ON TFT devices at V_GS_ = 0 V or a positive on-set voltage for p-type OTFT). Its advantage is its high noise margin, which allows more circuit robustness against process variations compared to diode-load logic design. The pseudo-CMOS inverter is composed of four p-type OTFTs (M1, M2, M3, and M4) with varied W/L ratios (Fig. 6a). The channel length of each OTFT was l5 μm, the channel widths for transistors M1, M2, M3, and M4 were 300, 300, 600, and 600 μm, respectively, and the gate-to-source and gate-to-drain overlap (L_OV_) was 30 μm. An image of the fabricated pseudo-CMOS inverter is shown in Fig. 6b. For static characterization of the fabricated inverter voltage transfer characteristics, the sweeping input voltage signals for different bias voltages (V_DD_ =  − V_SS_) between − 5 and − 20 V (Fig. 6c). Sharp switching characteristics with little hysteresis were obtained for the 4H–21DNTT OTFT-based inverter. The voltage gain is defined as ΔV_OUT_/ΔV_IN_ and it is estimated to be 31.57 and 32.1 at a supply voltage of − 20 V for glass and flexible substrates, respectively (flexible inverter characteristics are listed in Supplementary Information Fig. S11). The trip point (V_Trip_) of the inverter is defined as the voltage at which V_OUT_ = V_IN._ Trip points were estimated to be 16.3 V and 16.8 V at a supply voltage of − 20 V, for glass and flexible substrates, respectively. The ideal trip point of a pseudo-CMOS inverter is equal to half of the supply voltage (V_DD_/2).

Figure 6d shows the transient characteristics of the inverter on the glass substrate using a 30 V square wave input signal (V_in_) with a frequency of 5 kHz and a bias voltage (V_DD_ = V_SS_) of 20 V (flexible inverter Fig. S11). The average high-to-low and low-to-high propagation delays (τ_pd_) (Figs. S10, S13**)** of our inverter circuits were 6.9 μs and 18.8 μs, respectively. The rise (τ_r_) and fall (τ_f_) time constants were determined by fitting exponential functions to the measured transient output voltage signal. A rise time of 35 μs and a fall time of 29.8 μs were observed for glass substrate, while a rise time of 72.7 μs and a fall time of 59.4 μs were observed for flexible (PEN) substrate, for a supply voltage of 20 V (Figs. S9, S12).

### NAND logic circuit performance based on 4H–21DNTT OTFT

**Figure 7:**
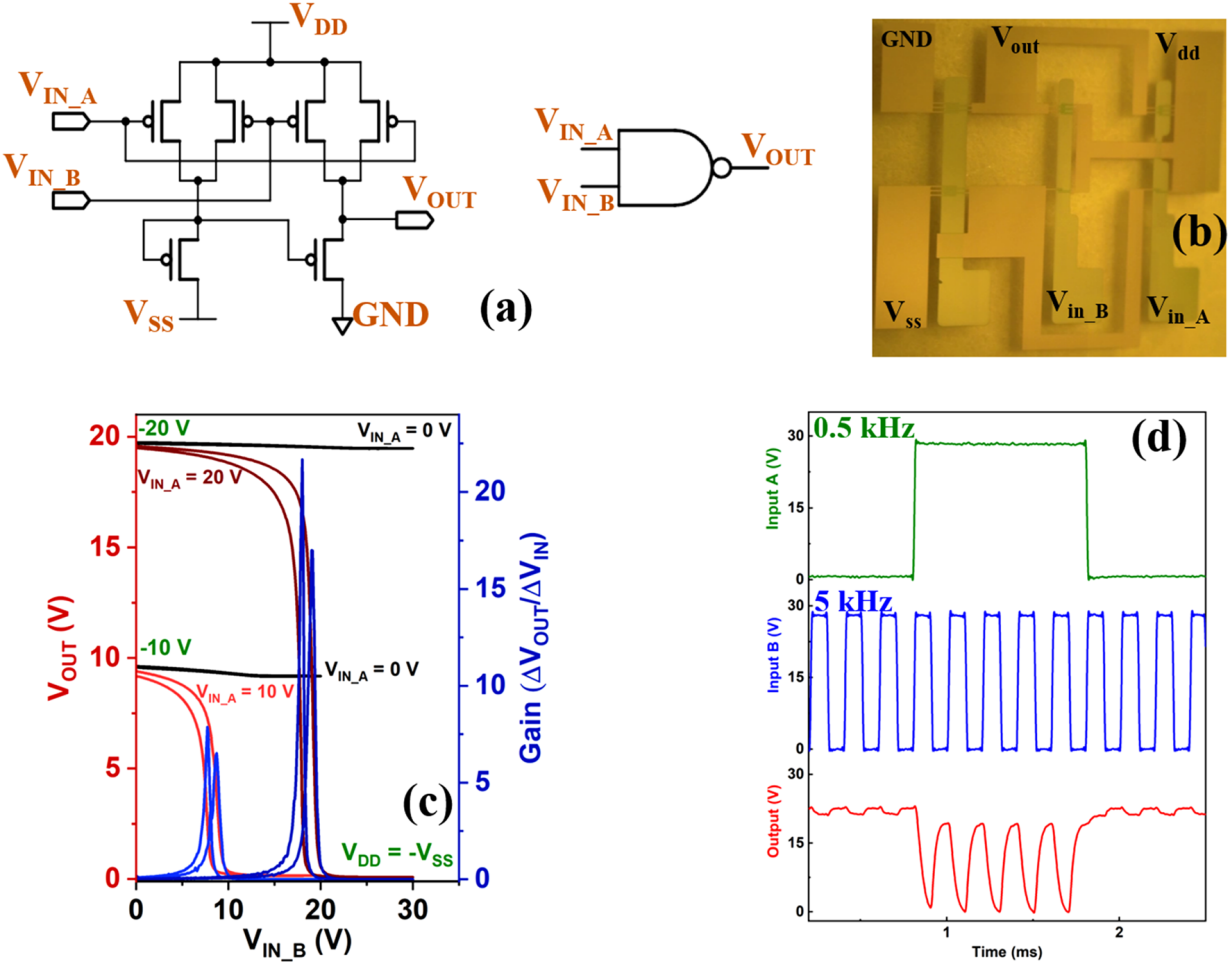
NAND logic circuit design and characteristics. (**a**) Circuit schematic of NAND device with zero-V_GS_-load p-type pseudo-CMOS design. (**b**) Photograph of fabricated NAND circuit. (**c**) Measured output voltage (V_OUT_) and small-signal gain as a function of input voltage B (V_IN_B_) for input voltage A V_IN_A_ = 0 V and V_IN_A_ = 10 V when V_DD_ = − 10 V and V_IN_A_ = 20 V when V_DD_ =  − 20 V. (**d**) Dynamic response of NAND logic circuit with input signals of frequencies, V_IN_A_ = 0.5 kHz and V_IN_A_ = 5 kHz.

To further demonstrate the potential of 4H–21DNTT OTFT and assess the reproducibility of our fabrication process, we studied the performance of a pseudo-CMOS based NAND circuit, which is a basic building block to configure D-FF, shift registers, and counters. A schematic of the pseudo-CMOS logic-based NAND circuit is shown in Fig. 7a. The NAND circuit is composed of six p-type OTFTs (T1, T2, T3, T4, T5, and T6) with various W/L ratios. The channel length of each OTFT was l5 μm, the channel widths for transistors T1, T2, T3, T4, T5, and T6 were 300, 300, 600, 600, 1200, and 1200 μm, respectively, and the gate-to-source and gate-to-drain overlap (L_OV_) was 30 μm. An image of the fabricated NAND circuit is shown in Fig. 7b. Figure 7c shows the static input–output voltage characteristics of NAND circuit (i) at a bias voltage of -20 V (V_SS_ =  − V_DD_) with input V_B_ swept linearly from 0 to 30 V for two V_A_ inputs of 0 V and 20 V, and (ii) at a bias voltage of − 10 V (tuning voltage V_SS_ =  − V_DD_) with input V_B_ swept linearly from 0 to 20 V for two V_A_ inputs of 0 V and − 10 V. At fixed V_A_ inputs of − 20 V and − 10 V, the pseudo-CMOS-based NAND circuit exhibited good switching characteristics with small hysteresis. The NAND circuit showed voltage gains of 21.7 and 22.6 at a supply voltage of − 20 V for glass and flexible substrates, respectively (see Fig. S16 Supplementary Information for flexible NAND characteristics). The trip points of the input V_A_ were 16.8 V and 16.3 V at a supply voltage of − 20 V for glass and flexible substrates, respectively, which is close to the ideal value of V_DD_/2. It can be seen from the static characteristics that when both inputs are logic high (V_A_ = 20/10 V and V_B_ ≥ 20/10 V), the output voltage is logic low (V_OUT_ < 0.2 V), and when the input V_A_ is low (V_A_ = 0 V), for both high- and low-input V_B_ (i.e., V_B_ = 0–30 V), the output voltage is always logic high (V_OUT_ > 0.2 V), demonstrating good NAND device characteristics.

The dynamic performance of the NAND logic circuit was evaluated by applying two square-wave input signals (V_A_ and V_B_) of amplitude 30 V; V_A_ with a frequency of 0.5 kHz and V_B_ with a frequency of 5 kHz (Fig. 7d, S16b). The average high-to-low and low-to-high propagation delays (τ_pd_) of the NAND circuits were measured to be 26.2 μs and 22.1 μs, respectively (Figs. S15, S18). Rise and fall times of 46.6 μs and 56.8 μs for glass substrate were observed for a supply voltage of 20 V (Figs. S14, S17). The combination of further reductions in contact resistance and an enhancement in mobility for short-channel OTFT devices is expected to yield even higher dynamic logic circuit performance.

## Discussion

In the presented work, we have successfully demonstrated 4H–21DNTT bottom-contact OTFTs fabricated on flexible (PEN) and glass substrates with a low contact resistance of 1 kΩcm, and we recorded a high mobility of 8.8 cm^2^ V^−1^ s^−1^ for a short channel length of 15 μm. High-quality 4H–21DNTT thin films with long single crystal domains on both flexible and glass substrates were obtained by temperature and speed-controlled solution shearing. We confirmed that high-quality OSC films in conjunction with an improved charge injection due to PFBT-treated Au electrodes resulted in lowering the contact resistance of the bottom-contact TFTs and enabled higher frequency operation in OTFT logic circuits. Furthermore, we fabricated a pseudo-CMOS architecture-based inverter and NAND logic circuit and obtained signal-propagation delays of 32.7 μs and 70.1 μs, respectively, at a bias voltage (V_DD_ =  − V_SS_) of 20 V, for bottom-contact 4H–21DNTT TFTs fabricated on flexible PEN substrates. We demonstrated an improvement in short-channel 4H–21DNTT OTFT mobility along with a reduction in contact resistance, which is essential for the operation of high-frequency logic circuits. The demonstration of such performance and its achievement using a bottom contact solution processed OTFT with thick gate dielectric presents an achievable pathway towards all-printed, low-cost, industrial production of high resolution OTFTs for driving flexible displays, sensor chips, and RFID applications. In conclusion, we used a very simple solution shearing fabrication method for achieving crystalline thin films of 4H–21DNTT organic semiconductor. High-performance OTFTs were demonstrated using these crystalline films with an intrinsic mobility of 15.1 cm^2^ V^−1^ s^−1^ and good environmental stability of their electrical performance. The short channel performance of 4H–21DNTT OTFTs on both rigid and flexible substrates is comparable to the best results obtained in literature. This basic study of 4H–21DNTT OTFT properties and its successful application in inverter and NAND integrated circuits demonstrates a promising potential of 4H–21DNTT OSC for applications in flexible, low-cost electronics with further improvement in the contact resistance of OTFTs.

## Methods

### Materials

The 2,3,4,5,6-pentafluorothiophenol (PFBT) and solvents were obtained from Sigma-Aldrich and were used without further purification. The organic semiconductor 4H–21DNTT was synthesized and purified and supplied by Ushio Chemix.

### OTFT and logic circuit fabrication

The glass substrates were sequentially cleaned prior to the use by ultrasonication with detergent, deionized water, acetone, and isopropanol for 10 min each and blown dried with nitrogen (99.99%) and the substrates were further cleaned with UV ozone for 10 min. The same method was used to fabricate all OTFTs and inverter and NAND logic circuits on glass and PEN substrates presented in this work. Aluminium (Al) gate electrodes (50 nm) were vacuum deposited continuously through a shadow mask onto the substrates. Parylene C gate dielectric (≈ 550 nm) was deposited on Al gate electrodes using chemical vapor deposition (CVD) system. Gold (Au) source-drain (S/D) electrodes (50 nm) were thermally deposited in vacuum through a shadow mask onto gate dielectric/gate substrate, to fabricate bottom-gate, bottom-contact configuration device. The Au electrodes were subsequently treated with 5 mM PFBT in 2-propanol to form a self-assembled monolayer (SAM) layer on Au electrodes. The OTFT channel width was in range of 380–400 μm, while channel length varied between 10 to 150 μm. Channel lengths for all inverter and NAND logic circuit devices were 15 μm. The PFBT monolayer improves the wetting property of Au during and substantially reduces the contact resistance value and improves OTFT performance. Finally, 6,6 bis (trans-4-butylcyclohexyl)-dinaphtho[2,1-b:2,1-f]thieno[3,2-b]thiophene (4H–21DNTT) organic semiconductor thin film was solution coated using a home-built solution shearing setup as shown in Fig. 1d. The substrate is kept on a hot plate with temperature 90 ℃ and solution was injected into the gap space ≈ 100 μm between blade and substrate. To fabricate large crystalline film of 4H–21DNTT with no gaps, temperature, solute concentration and blade scanning speed were optimized such that the solution drying speed is approximately the same as scanning speed (Fig. 2). During the solution shearing process, the same volume of solution was injected in the gap for each transistor in order to keep the meniscus shape constant which is important in achieving good reproducibility and less variation in the OTFT performance parameters.

### Thin film characterization and OTFT electrical measurements

Film thickness and surface morphology were measured using AFM (SPI3800N SPA 500 AFM probe station)**.** The out-of-plane XRD analysis of the 4H–21DNTT films was performed on a Rigaku Smartlab XRD with 9 kW X-ray power and the sample was scanned for 2θ values ranging from 0° to 25°. The Au work function and 4H–21DNTT HOMO level were measured using Riken Keiki AC3 photoelectron spectrophotometer in air (PESA). Crystal orientation and uniformity were characterized by a polarized optical microscope (Nikon Eclipse LV100ND). The OTFT and logic circuit device fabrication, storage and measurement were performed at ambient condition (relative humidity 65% and with controlled temperature ranging from 25 to 27 ℃). The transfer and output characteristics of the OTFT and logic circuit devices were measured at room temperature in air with a Keithley 4200 semiconducting parameter analyzer. Field‐effect mobility was calculated in the saturation (*V*_D_ =  − 20 V) and linear regime (*V*_D_ =  − 1 V) from the linear fitting to plots of $\sqrt {I_{D} }$ versus *V*_G_ and *I*_D_ versus *V*_G,_ respectively using following equations:

3 $${\text{Saturation Regime}}:{ }\sqrt {I_{D} } = \sqrt {\mu_{sat} } \sqrt {\frac{{WC_{i} }}{2L}} \left( {V_{G} - V_{TH} } \right),$$

4 $${\text{Linear Regime}}:{ }I_{D} = \mu_{lin} \frac{{WC_{i} }}{L}\left( {\left( {V_{G} - V_{TH} } \right)V_{D} } \right).$$

The reliability factor was calculated using following equation^40^,

5 $$r = \frac{{\mu_{effective} }}{{\mu_{claimed} }} = \left( {\frac{{\sqrt {\left| {I_{D}^{max} } \right|} - \sqrt {\left| {I_{D}^{0} } \right|} }}{{\left| {V_{G}^{max} } \right|}}} \right)^{2} /\left( {\frac{{\partial \sqrt {\left| {I_{D} } \right|} }}{{\partial V_{G} }}} \right)_{claimed}^{2} .$$

A Tektronix AFG1022 function generator, a Tektronix TBS1104 digital oscilloscope, and an Agilent 33502A amplifier were used to assess the transient performance of the inverters and NAND logic circuits.

## Supplementary Information

Supplementary Information.
