# Supplementary material for: Flexible, high mobility short-channel organic thin film transistors and logic circuits based on 4H–21DNTT
Source: Sci Rep. 2021 Jun 3;11:11710. doi: 10.1038/s41598-021-91239-7 (PMC8175738; doi:10.1038/s41598-021-91239-7)
Supplement: Supplementary file 1 — Supplementary Information. [file 41598_2021_91239_MOESM1_ESM.pdf]

Supplementary Information

Flexible, high mobility short-channel organic thin film transistors and logic circuits based on 4H-21DNTT

Anubha Bilgaiyan[1,*], Seung-Il Cho[1], Miho Abiko[1], Kaori Watanabe[1] and Makoto Mizukami[1*]

[1]Innovation Center for Organic Electronics, Yamagata University 1-808-48, Arcadia, Yonezawa, Yamagata, 992-0119, Japan
*anubha.bilgaiyan@yz.yamagata-u.ac.jp (A.B.)
* m_mizukami@yz.yamagata-u.ac.jp (M.M.)

Figure S1 Absorption spectra of solution sheared thin film of 4H-21DNTT

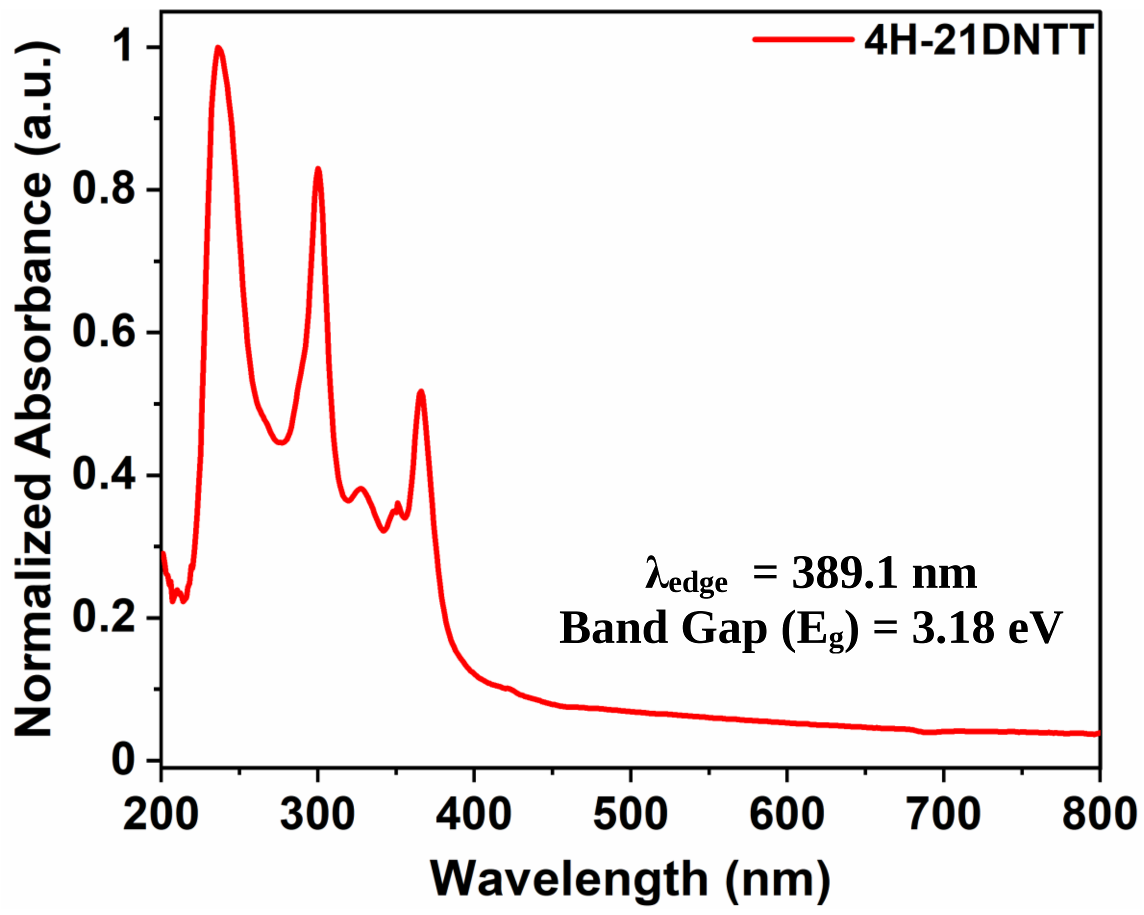

Figure S2 Transfer characteristics of 4H-21DNTT bottom-gate, bottom-contact OTFT with Parylene C as gate dielectric for different depostition conditions of 4H-21DNTT: a) v_{scan} = 32 μms^{-1} & Conc.: 0.1 wt%, b) v_{scan} = 20.5 μms^{-1} & Conc.: 0.1 wt%, c) v_{scan} = 12.5 μms^{-1} & Conc.: 0.1 wt%, d) v_{scan} = 32 μms^{-1} & Conc.: 0.09 wt%, e) v_{scan} = 20.5 μms^{-1} & Conc.: 0.09 wt% , f) v_{scan} = 12.5 μms^{-1} & Conc.: 0.09 wt%.

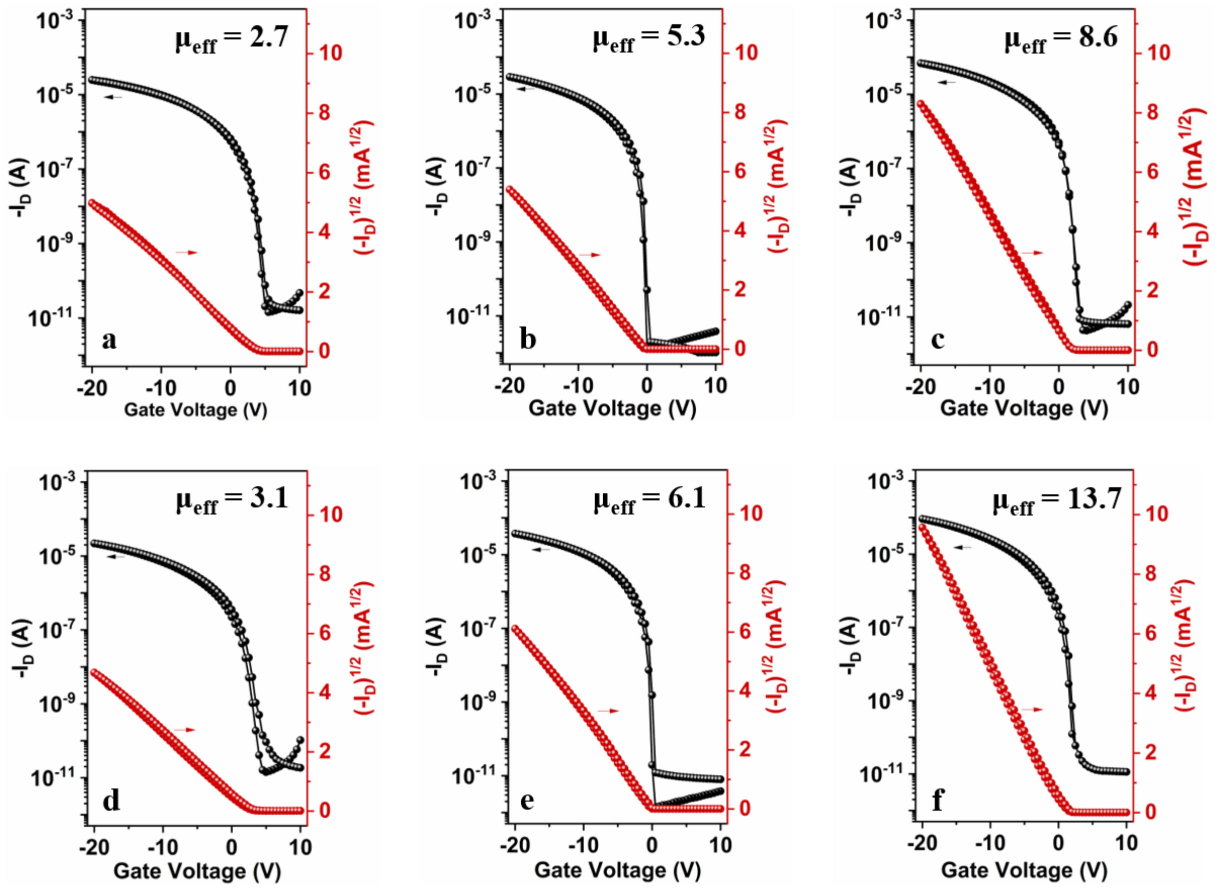

Figure S3 AFM images of 4H-21DNTT deposited by solution shearing (blade scanning speed 12.5 μms^{-1}) for two 4H-21DNTT concentrations a) 0.09 wt% film thickness was around 9 nm coreesponding to 3 monolayers. b) 0.1 wt % thick crystals upto 50 nm wwere observed.

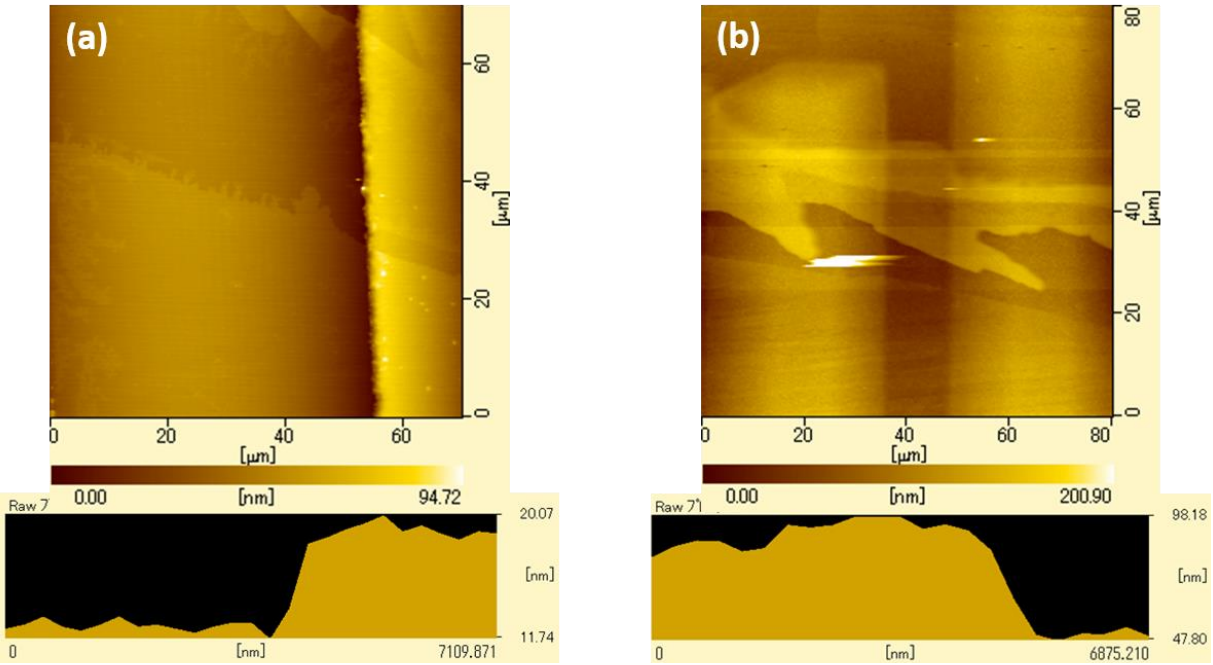

Figure S4 Transfer characteristics of 4H-21DNTT bottom-gate, bottom-contact OTFT with Parylene C as gate dielectric for channel length ranging from 10 - 120 μm.

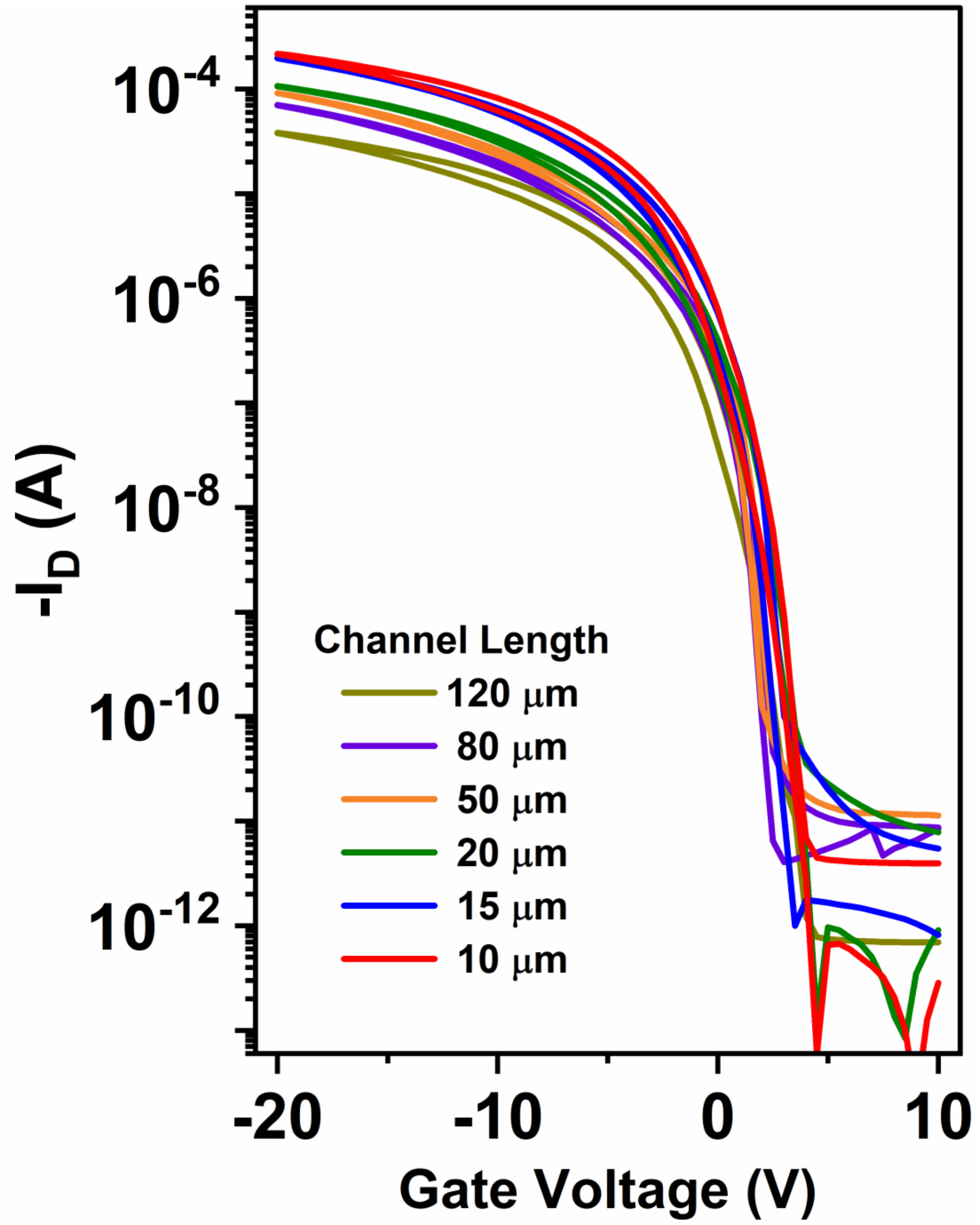

Figure S5 Statistical Variation of saturation mobility (μ_{sat}) for 4H-21DNTT OTFTs.

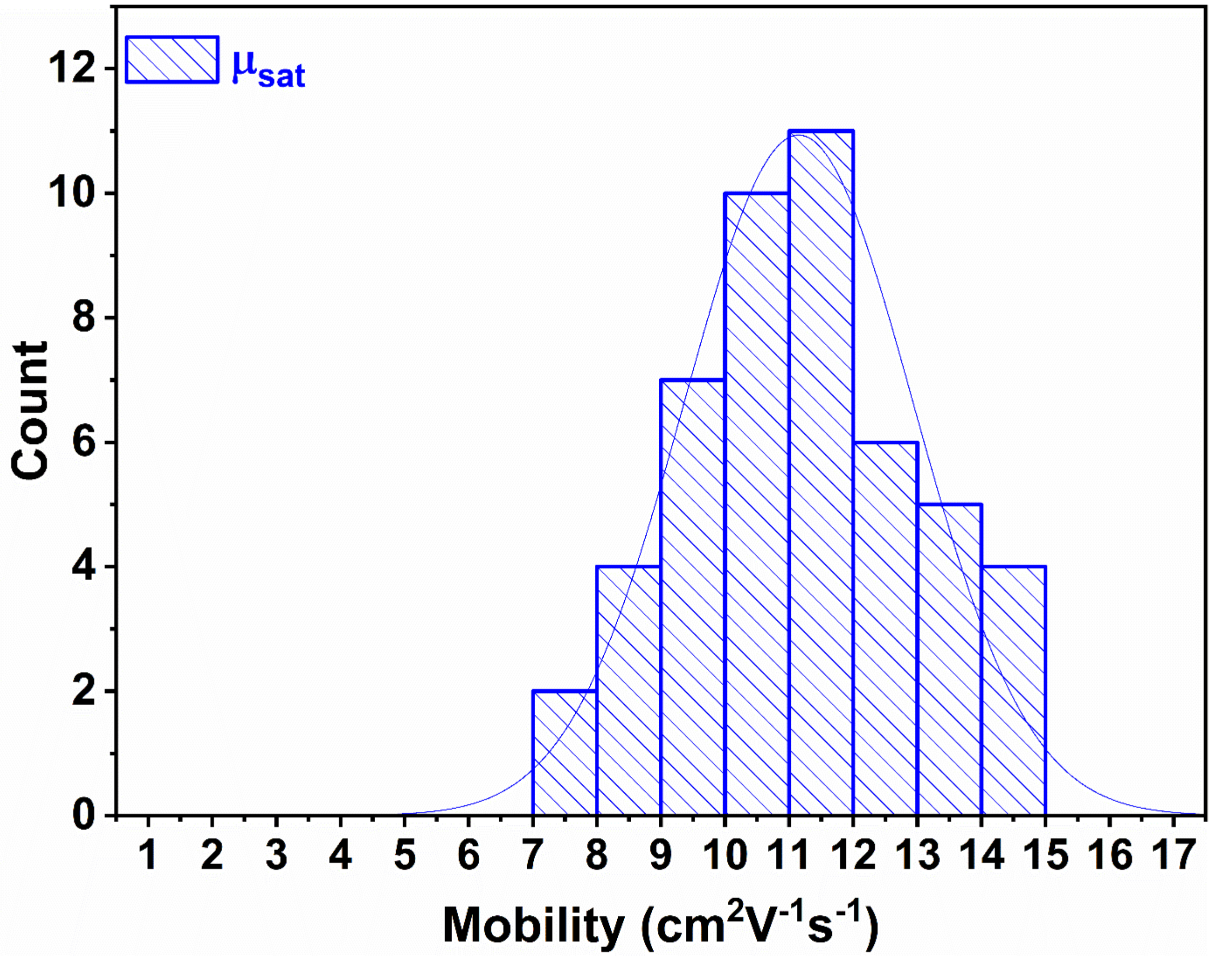

Figure S6 **Literature Overview:** (a) Comparision of the reported width-normalized contact resistance (R_CW) in coplanar OTFTs. (References: [1-18] in supporting information) (b) Comparision of the reported intrinsic mobility (μ_o) normalized by squared channel length (L^2) (as OTFT transit frequencies ($f_T \propto \mu_o / L^2$) for OTFTs. (References: [12-18, 39-59] in main manuscript.)

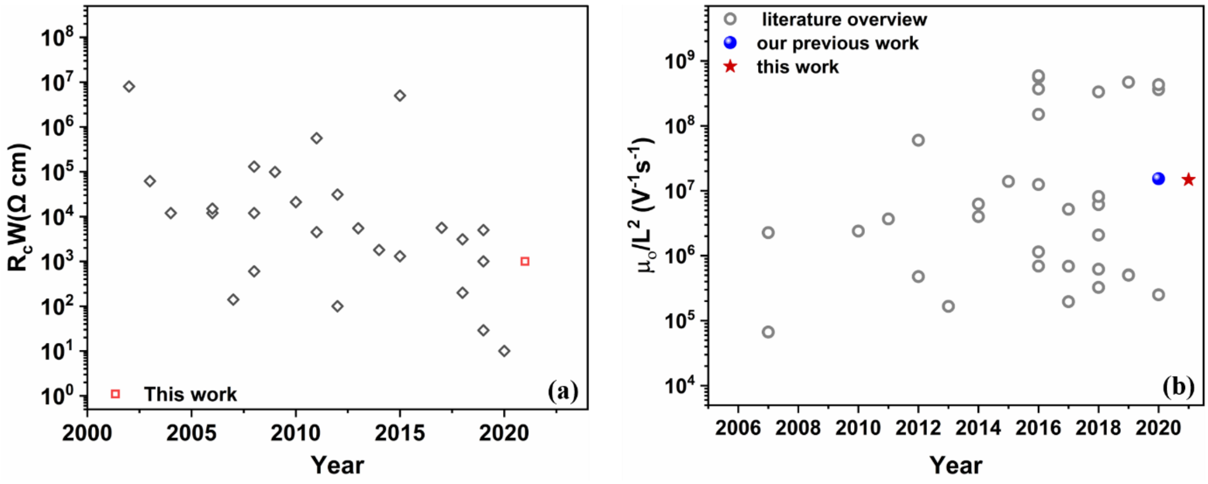

Figure S7 4H-21DNTT OTFT Contact resistance analysis for contacts without PFBT treatment: (a) Total device resistance ($R_{TOTAL}W$) plotted as a function of the channel length for OTFT for various gate overdrive voltages ($V_G - V_{TH}$). (b) Contact resistance calculated using the transmission line method (TLM) plotted as a function of gate overdrive voltages ($V_G - V_{TH}$).

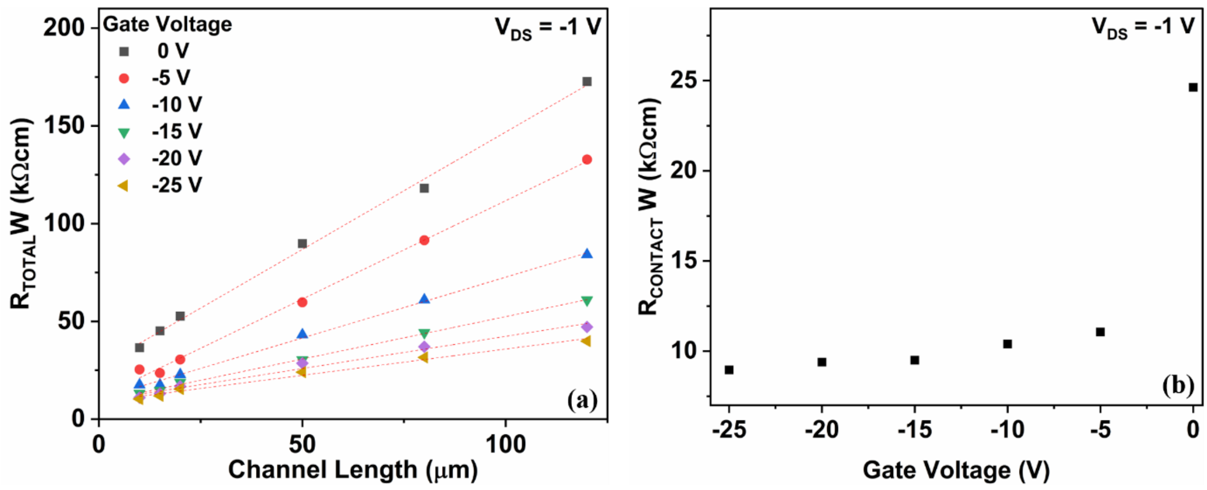

Figure S8 Stability Analysis: Transfer characteristics of 4H-21DNTT OTFTs on the Parylene C/glass substrate: black traces, freshly fabricated device; blue traces, after 30 days storage in ambient conditions (relative humidity 65% and with controlled temperature ranging from 25 ℃ to 27 ℃).

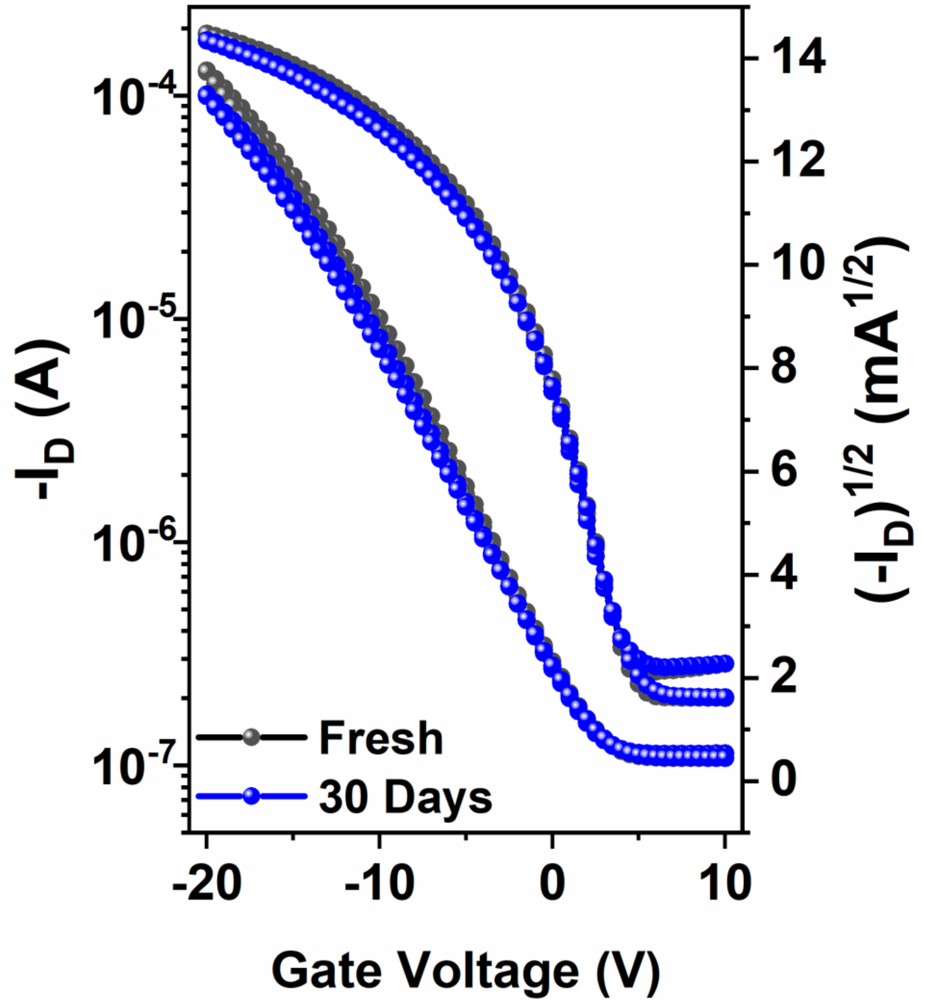

Figure S9 Zero-V_{GS}-load p-type pseudo-CMOS inverter (glass substrate) dynamic output characteristics: Calculation of rise time (τ_r i.e., when output signal changes from low to high) and fall time (τ_f i.e., when output signal changes from high to low)

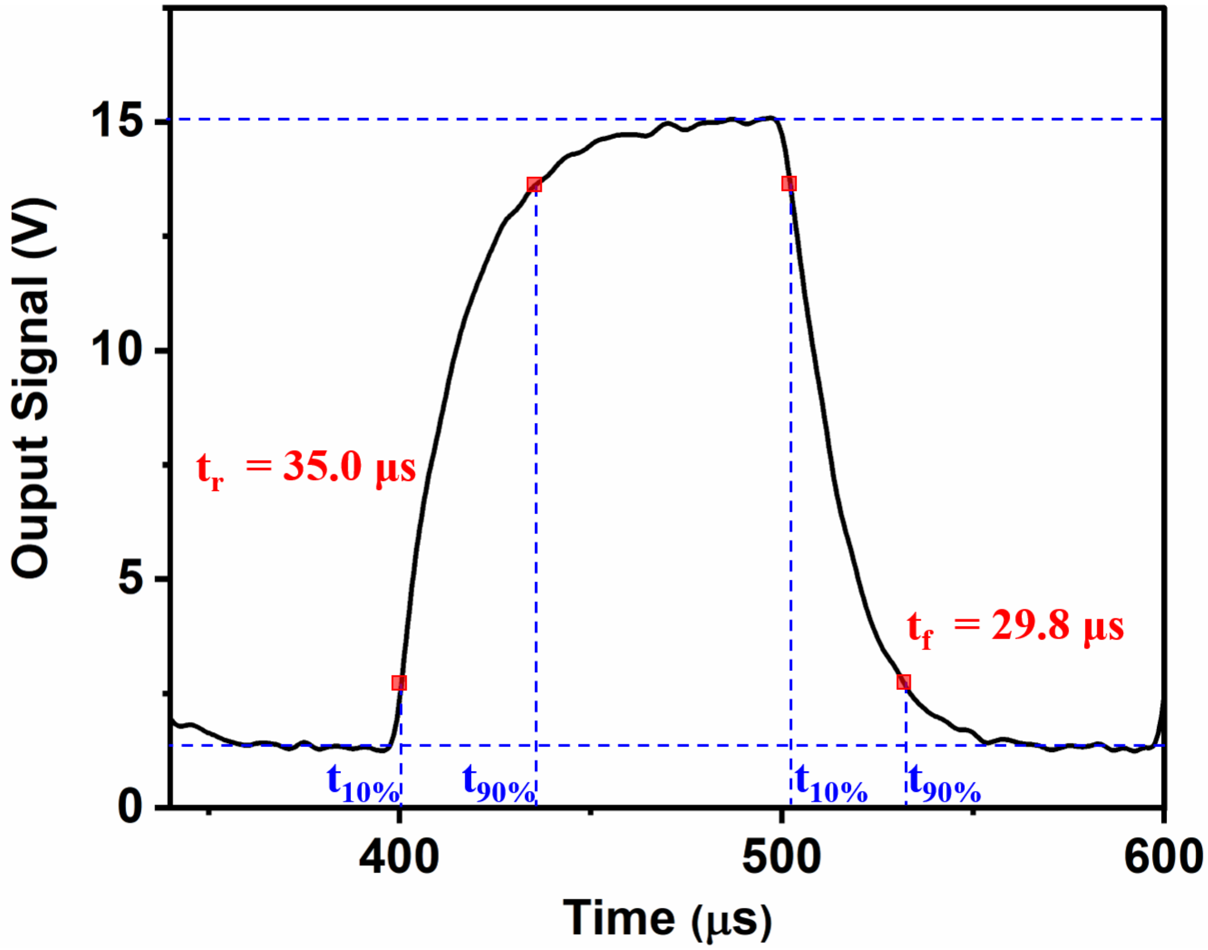

Figure S10 Zero-V_{GS}-load p-type pseudo-CMOS inverter (glass substrate) dynamic output characteristics: Calculation of propagation delay (τ)

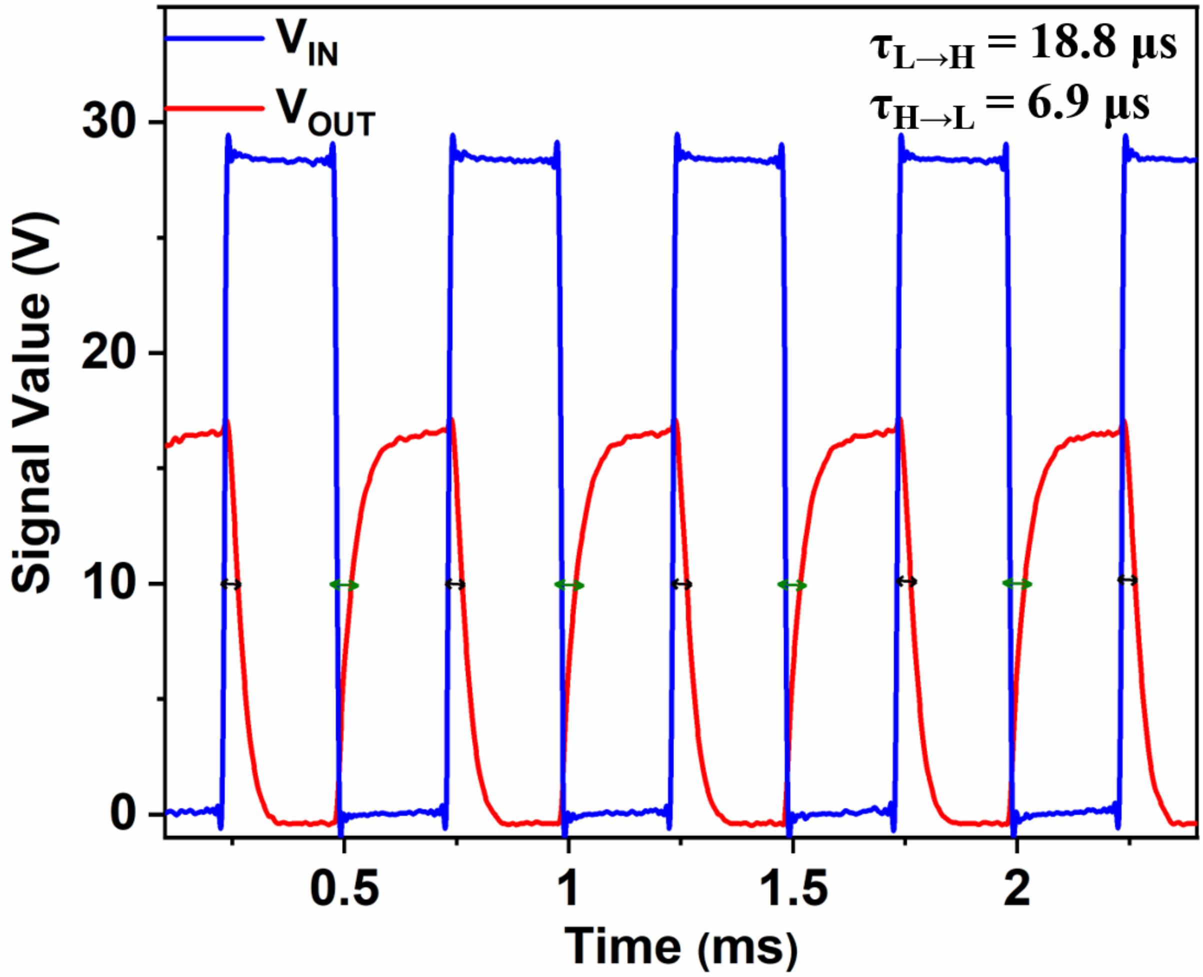

Figure S11 Zero-V_{GS}-load p-type pseudo-CMOS inverter (flexible PEN substrate) output characteristics (a) Measured output voltage (V_{OUT}) and small-signal gain as a function of input voltage (V_{IN}) for supply voltages (V_{DD}) between -10 and -20 V. (b) Dynamic response of zero-V_{GS}-load p-type pseudo-CMOS inverter with input signal frequency of 1 kHz

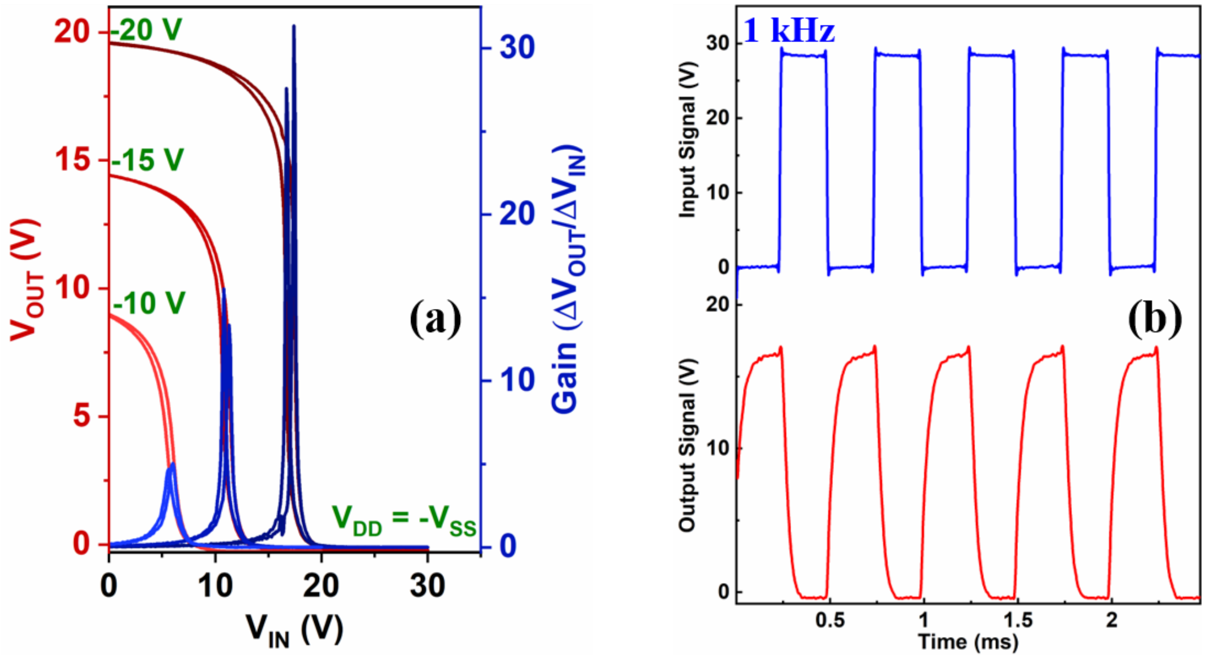

Figure S12 Zero-V_{GS}-load p-type pseudo-CMOS inverter (flexible PEN substrate) dynamic output characteristics: Calculation of rise time (τ_r i.e., when output signal changes from low to high) and fall time (τ_f i.e., when output signal changes from high to low)

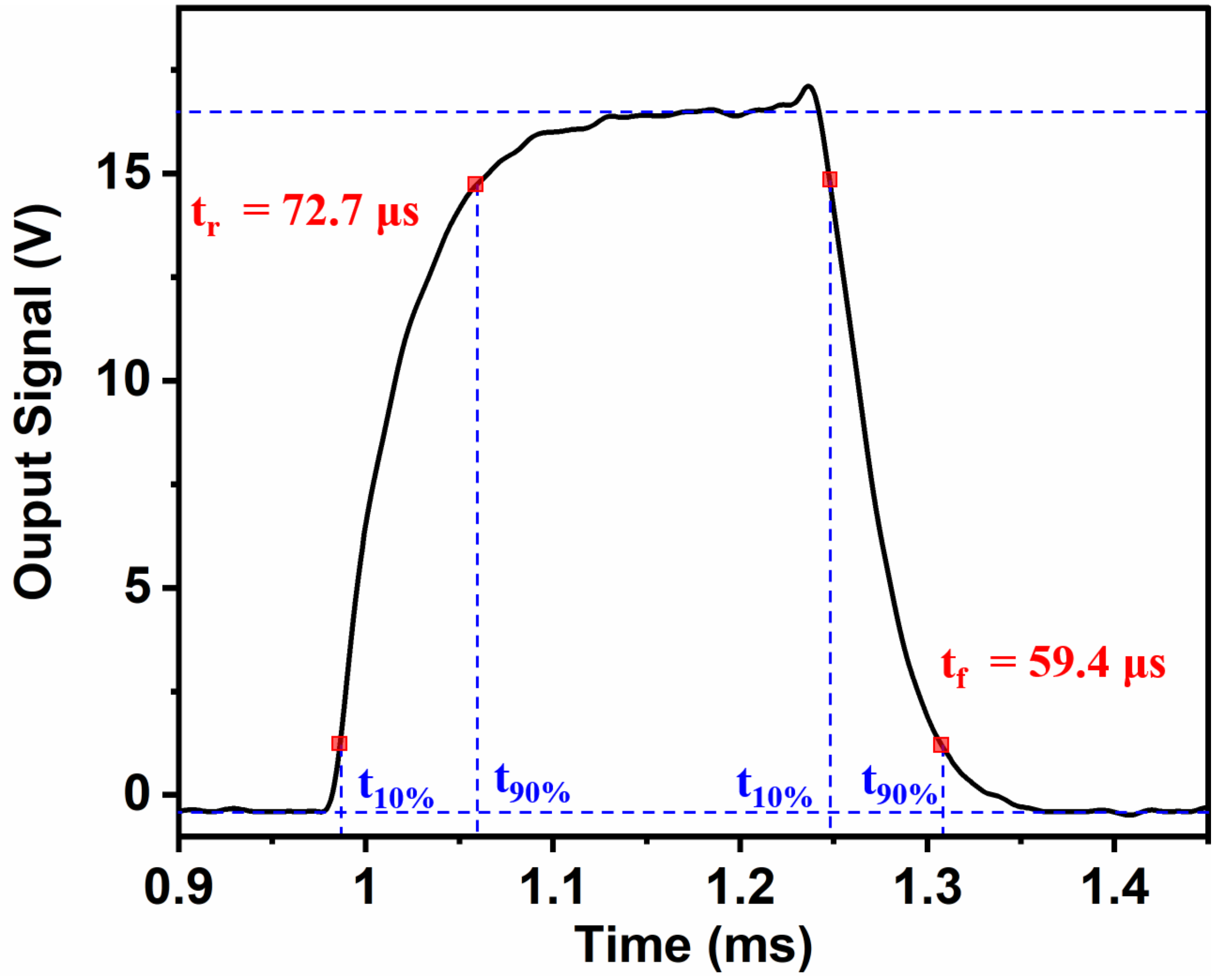

Figure S13 Zero-V_{GS}-load p-type pseudo-CMOS inverter (flexible PEN substrate) dynamic output characteristics: Calculation of propagation delay (τ).

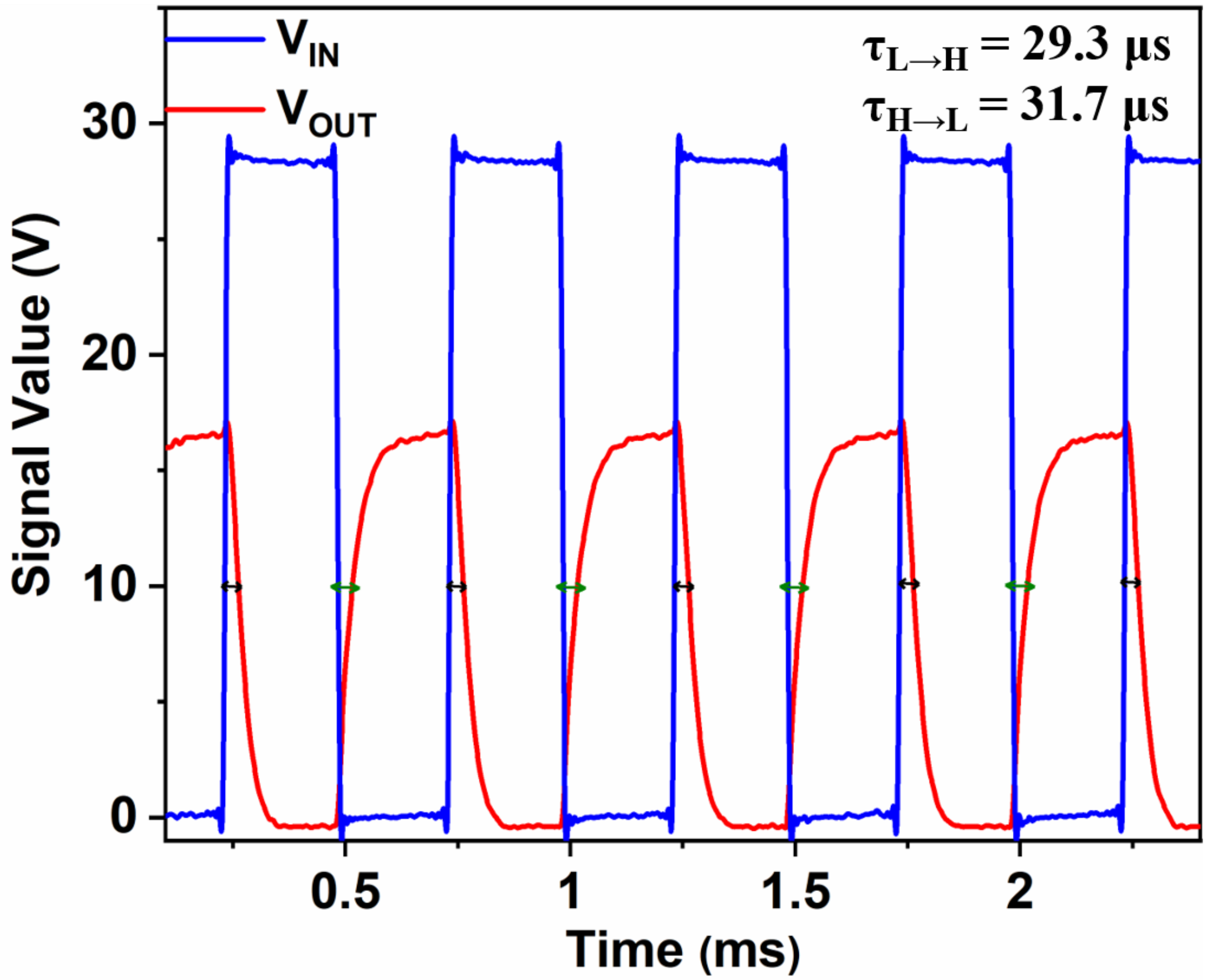

Figure S14 NAND logic circuit based on zero-V_{GS}-load p-type pseudo-CMOS design (glass substrate) dynamic output characteristics: Calculation of rise time (τ_r i.e., when output signal changes from low to high) and fall time (τ_f i.e., when output signal changes from high to low)

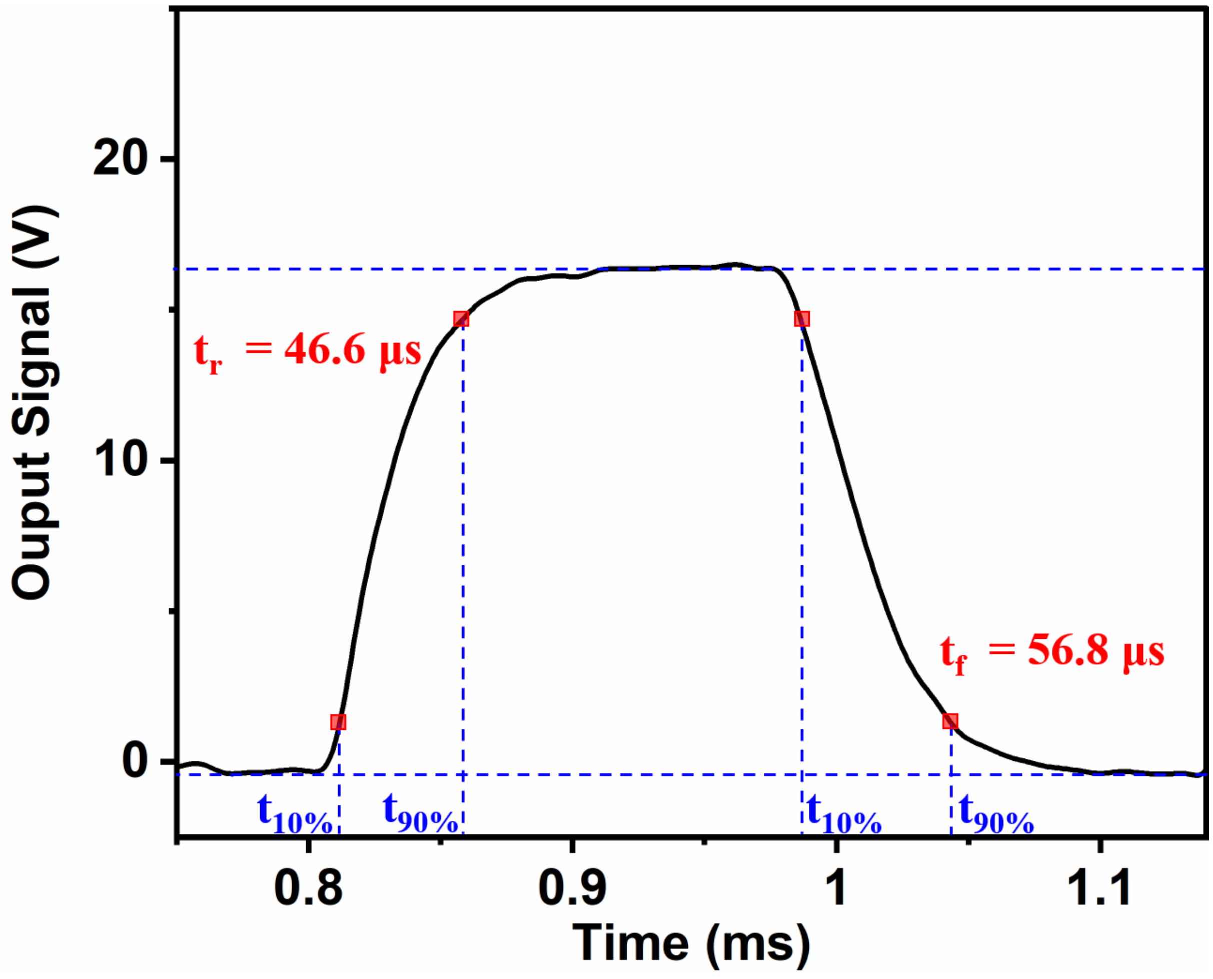

Figure S15 NAND logic circuit based on zero-V_{GS}-load p-type pseudo-CMOS design (glass substrate) dynamic output characteristics: Calculation of propagation delay (τ).

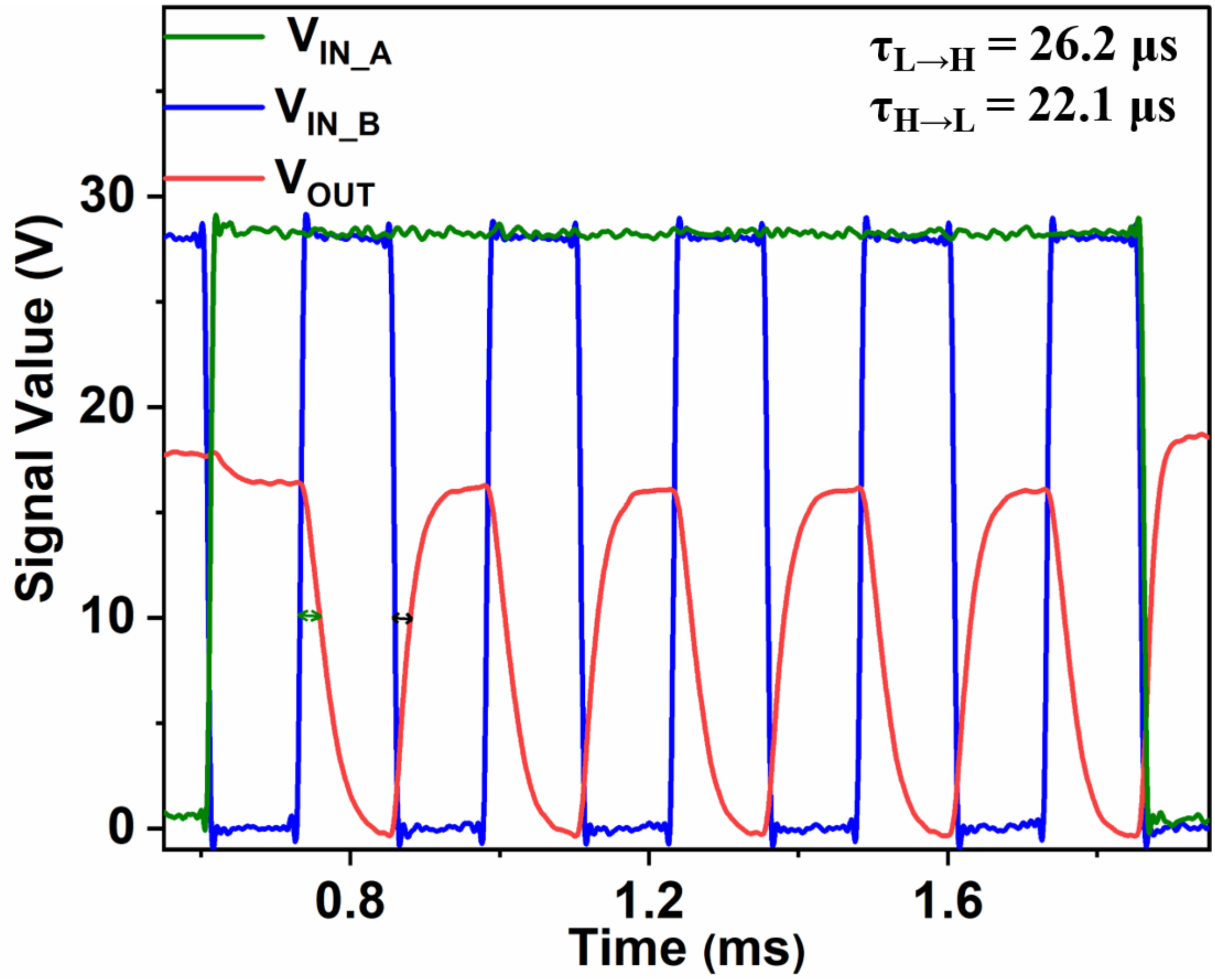

Figure S16 NAND logic circuit based on zero-V_{GS}-load p-type pseudo-CMOS design (flexible PEN substrate) output characteristics (a) Measured output voltage (V_{OUT}) and small-signal gain as a function of input voltage (V_{IN}) for supply voltages (V_{DD}) between -10 and -20 V. (b) Dynamic response of NAND logic circuit with input signals of frequencies V_{IN_A} = 150 Hz and V_{IN_B} = 1.5 kHz.

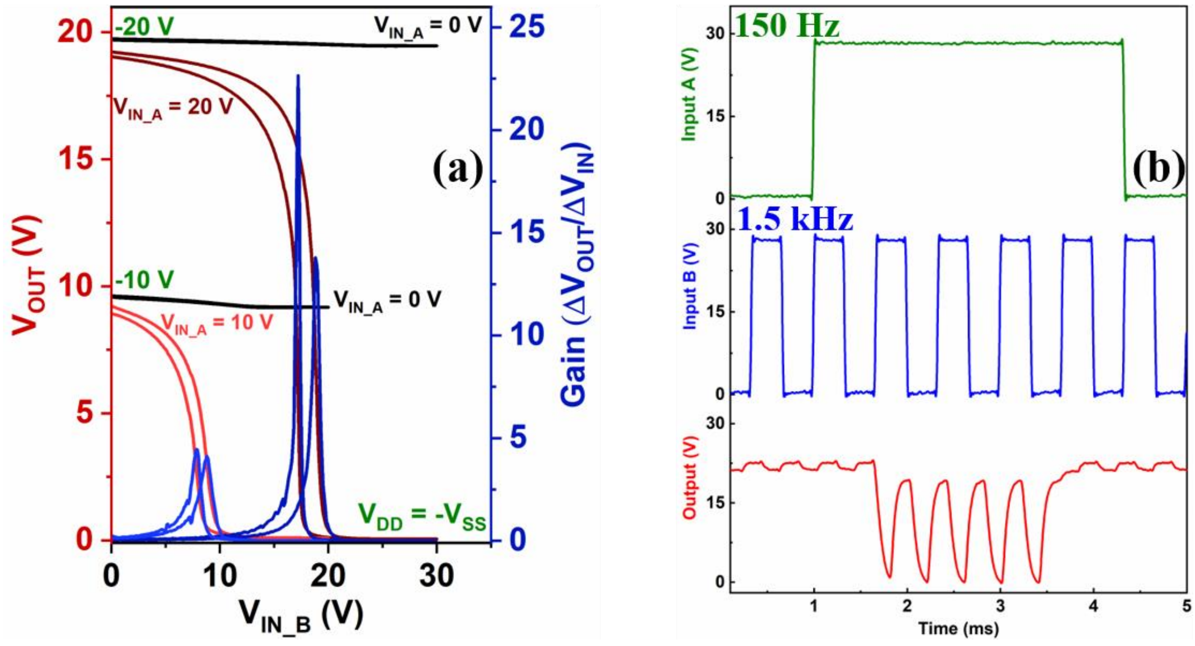

Figure S17 NAND logic circuit based on zero-V_{GS}-load p-type pseudo-CMOS design (glass substrate) dynamic output characteristics: Calculation of rise time (τ_r i.e., when output signal changes from low to high) and fall time (τ_f i.e., when output signal changes from high to low)

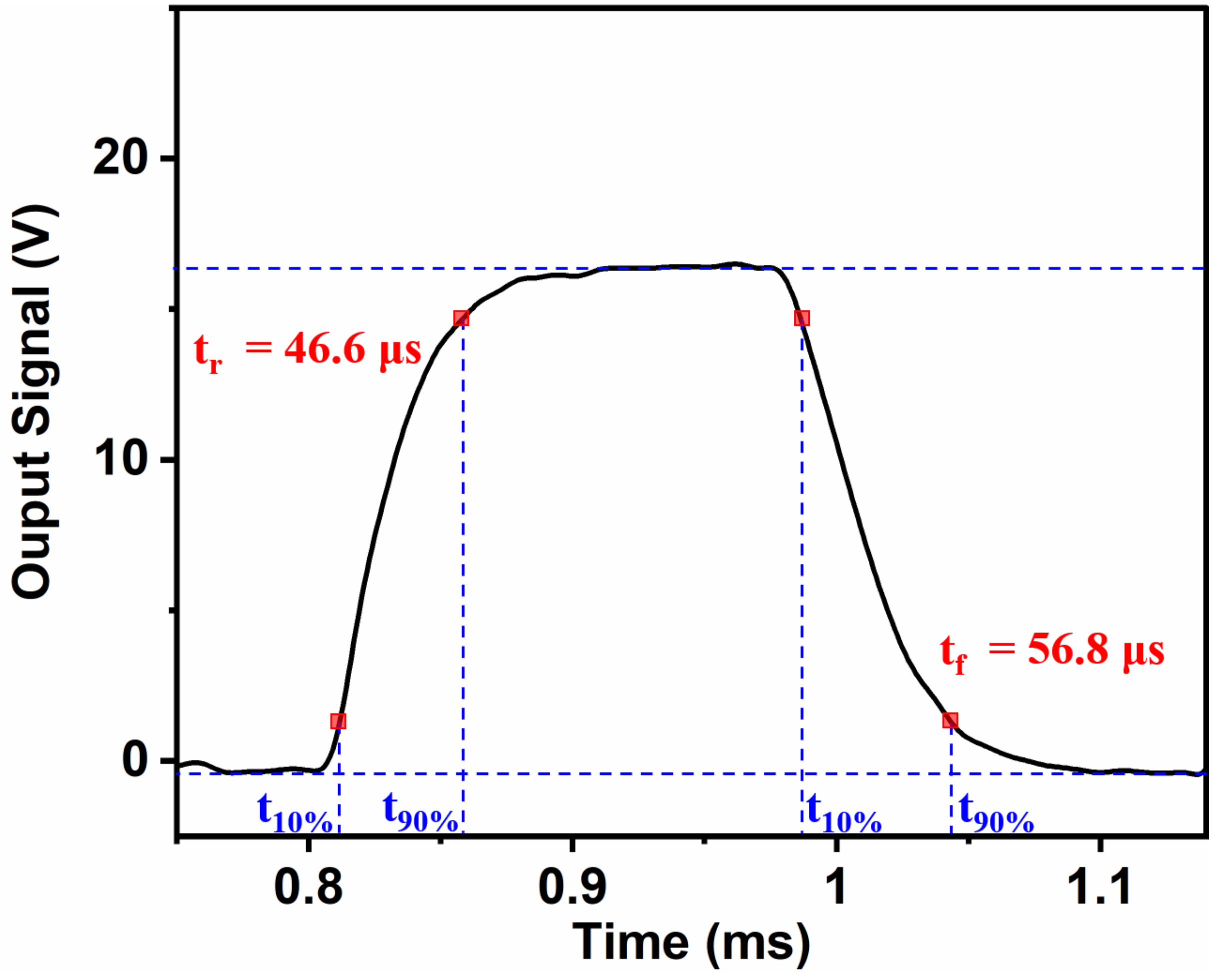

Figure S18 NAND logic circuit based on zero-V_{GS}-load p-type pseudo-CMOS design (flex PEN substrate) dynamic output characteristics: Calculation of propagation delay (τ).

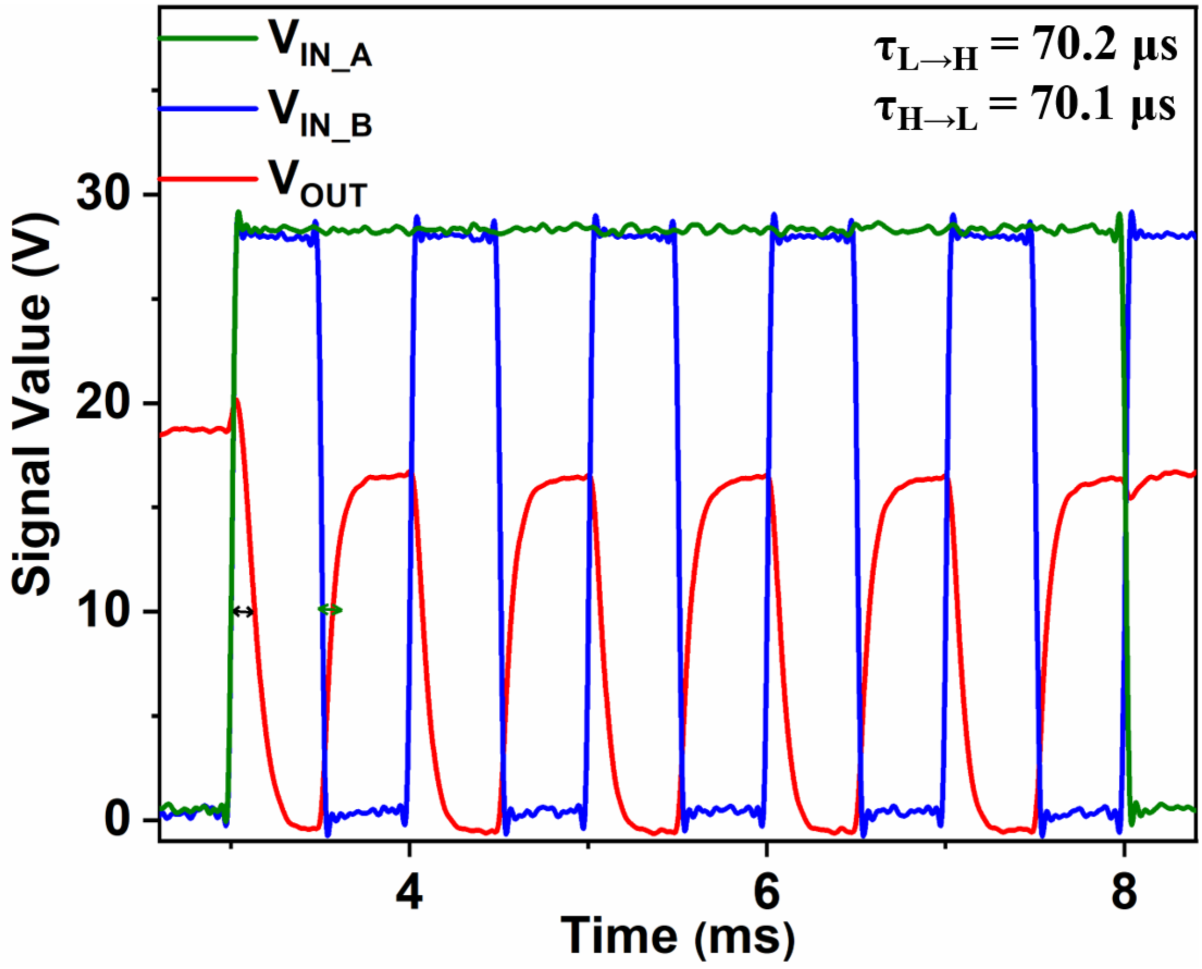

Table S1 OTFT circuit dynamic performance: Comparision of propgation delay for inverter based circuits with solution processed OTFT.

Parameters	[1]	[2]	[3]	[4]	[5]	This work
L (µm)	NA	21	5	29	5	15
OSC Deposition Method	Edge casting	Inkjet & Dispenser	Inkjet	Inkjet & Dispenser	Zone casting	Solution shearing
Circuit design schematic	CMOS	CMOS	Pseudo-CMOS	CMOS	Pseudo-CMOS	Pseudo-CMOS
H→L Propagation Delay (ms)	0.025 (D Flip Flop)	1.9 (Inverter) 2.7 (NAND)	0.16 (Inverter) 1.5 (NAND)	0.34 (Inverter)	0.042 (Inverter)	0.028 (Inverter) 0.068 (NAND)
L→H Propagation Delay (ms)	NA	1.2 (Inverter) 1.3 (NAND)	0.11 (Inverter) 0.1 (NAND)	NA	NA	0.023 (Inverter) 0.063 (NAND)

[1] Uno, M. et al. High-Yield, Highly Uniform Solution-Processed Organic Transistors Integrated into Flexible Organic Circuits. *Adv. Electron. Mater.* 2017 3 1600410

[2] Hayasaka, K. et al. Compact Organic Complementary D-Type Flip-Flop CircuitsFabricated with Inkjet Printing. *Adv. Electron. Mater.* 3, 1700208 (2017).

[3] Cho, S.-I. et al. Implementation of Logic Gates Using Organic Thin Film Transistor for Gate Driver of Flexible Organic Light-Emitting Diode Displays. *The journal of KIECS*, 14, 1, 87-95 (2019). DOI:10.13067/JKIECS.2019.14.1.87

[4] Takeda, Y. et al. Printed Organic Complementary Inverter with Single SAM Process Using a p-type D-A Polymer Semiconductor. *Appl. Sci.* 8, 1331 (2018)

[5] Janneck R. et al. Integration of highly crystalline C8-BTBT thin-films into simple logic gates and circuits. *Org. Electron.* 67, 64-71 (2019).

References

1. Street, R.A. & Salleo, A. Contact effects in polymer transistors. Appl. Phys. Lett. 81, 2887 (2002).
2. Yun, H. et al. Stencil Nano Lithography Based on a Nanoscale Polymer Shadow Mask: Towards Organic Nanoelectronics. Sci Rep 5, 10220 (2015).
3. Puntambekar, K.P., Pesavento, P. V., & Frisbie, C. D. Surface potential profiling and contact resistance measurements on operating pentacene thin-film transistors by Kelvin probe force microscopy. Appl. Phys. Lett. 83, 5539 (2003).
4. Borchert, J. W. et al. Small contact resistance and high-frequency operation of flexible low-voltage inverted coplanar organic transistors. Nat. Commun. 10, 1119 (2019).
5. Blanchet, G.B., Fincher, C. R., Lefenfeld, M. & Rogers, J. A. Contact resistance in organic thin film transistors. Appl. Phys. Lett. 84, 296 (2004).
6. Stadlober B., Orders-of-Magnitude Reduction of the Contact Resistance in Short-Channel Hot Embossed Organic Thin Film Transistors by Oxidative Treatment of Au-Electrodes. Adv. Funct. Mater. 17, 2687-2692 (2007).
7. Gundlach, D.J.et al. An experimental study of contact effects in organic thin film transistors. J. Appl. Phys. 100, 024509 (2006).
8. Hamadani, B.H. et al. Insights into the characterization of polymer-based organic thin-film transistors using capacitance-voltage analysis. Appl. Phys. Lett. 92, 203303 (2008).
9. Kim, H. et al. Laser forward transfer of silver electrodes for organic thin-film transistors. Appl. Phys. A 96, 441–445(2009).
10. Singh, K.A. et al. Effect of Self-Assembled Monolayers on Charge Injection and Transport in Poly(3-hexylthiophene)-Based Field-Effect Transistors at Different Channel Length Scales. ACS Appl. Mater. Interfaces. 3 ,8, 2973-2978 (2011).

11. Darmawan, P. et al. Optimal Structure for High-Performance and Low-Contact-Resistance Organic Field-Effect Transistors Using Contact-Doped Coplanar and Pseudo-Staggered Device Architectures. Adv. Funct. Mater. 22, 4577-4583 (2012).
12. Youn, J. et al. Influence of Thiol Self-Assembled Monolayer Processing on Bottom-Contact Thin-Film Transistors Based on n-Type Organic Semiconductors. Adv. Funct. Mater. 22, 1856-1869 (2012).
13. Singh, S. et al. Reduction of contact resistance by selective contact doping in fullerene n-channel organic field-effect transistors. Appl. Phys. Lett. 102, 153303 (2013).
14. Fukuda, K. et al. Fully Solution-Processed Flexible Organic Thin Film Transistor Arrays with High Mobility and Exceptional Uniformity. Sci. Rep. 4, 3947 (2015).
15. Janneck, R. et al. Integration of highly crystalline C8-BTBT thin-films into simple logic gates and circuits. Org. Electron. 67, 64-71 (2019).
16. Boudinet, D. et al. Modification of gold source and drain electrodes by self-assembled monolayer in staggered n- and p-channel organic thin film transistors. Org. Electron. 11, 227–237 (2010).
17. Waldrip, M. et al. Contact Resistance in Organic Field-Effect Transistors: Conquering the Barrier. Adv. Funct. Mater. 30, 1904576 (2020).
18. Liu, C., Xu, Y. & Noh, Y.-Y. Contact engineering in organic field-effect transistors. *Mater. Today* 18, 2, 79-96 (2015).
